# Polymer-induced biofilms for enhanced biocatalysis†

**DOI:** 10.1039/d2mh00607c

**Published:** 2022-07-21

**Authors:** Pavan Adoni, Andrey Romanyuk, Tim W. Overton, Paco Fernandez-Trillo

**Affiliations:** School of Chemistry, University of Birmingham, Edgbaston Birmingham B15 2TT UK f.fernandez-trillo@bham.ac.uk; School of Chemical Engineering, University of Birmingham, Edgbaston Birmingham B15 2TT UK t.w.overton@bham.ac.uk; Institute of Microbiology and Infection, University of Birmingham, Edgbaston Birmingham B15 2TT UK; Departamento de Química, Facultade de Ciencias and Centro de Investigacións Científicas Avanzadas (CICA), Universidade da Coruña 15071 A Coruña Spain

## Abstract

The intrinsic resilience of biofilms to environmental conditions makes them an attractive platform for biocatalysis, bioremediation, agriculture or consumer health. However, one of the main challenges in these areas is that beneficial bacteria are not necessarily good at biofilm formation. Currently, this problem is solved by genetic engineering or experimental evolution, techniques that can be costly and time consuming, require expertise in molecular biology and/or microbiology and, more importantly, are not suitable for all types of microorganisms or applications. Here we show that synthetic polymers can be used as an alternative, working as simple additives to nucleate the formation of biofilms. Using a combination of controlled radical polymerization and dynamic covalent chemistry, we prepare a set of synthetic polymers carrying mildly cationic, aromatic, heteroaromatic or aliphatic moieties. We then demonstrate that hydrophobic polymers induce clustering and promote biofilm formation in MC4100, a strain of *Escherichia coli* that forms biofilms poorly, with aromatic and heteroaromatic moieties leading to the best performing polymers. Moreover, we compare the effect of the polymers on MC4100 against PHL644, an *E. coli* strain that forms biofilms well due to a single point mutation which increases expression of the adhesin curli. In the presence of selected polymers, MC4100 can reach levels of biomass production and curli expression similar or higher than PHL644, demonstrating that synthetic polymers promote similar changes in microbial physiology than those introduced following genetic modification. Finally, we demonstrate that these polymers can be used to improve the performance of MC4100 biofilms in the biocatalytic transformation of 5-fluoroindole into 5-fluorotryptophan. Our results show that incubation with these synthetic polymers helps MC4100 match and even outperform PHL644 in this biotransformation, demonstrating that synthetic polymers can underpin the development of beneficial applications of biofilms.

## Abstract 2

Here, we show how synthetic polymers, prepared by a combination of controlled radical polymerization and in-situ screening, can be used to address a biological challenge – how to improve biofilm formation – to provide solutions to a field – biocatalysis – of industrial and societal relevance.

## Graphical abstract

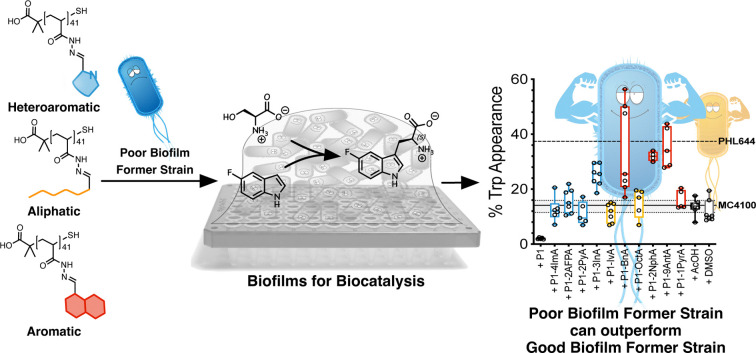

**New concepts**

We present in the attached manuscript a new materials-based methodology to induce biofilms in beneficial bacteria. Our solution relies on synthetic polymers, that work as simple additives during microbial culture, to promote biofilm formation. We have also demonstrated that these polymer-induced biofilms can increase the biocatalytic activity of *E. coli*, a workhorse in biotechnology. To the best of our knowledge, currently there are no methods that provide this simplicity and versatility when promoting biofilms for beneficial bacteria. Traits for biofilms formation are often introduced in bacteria through gene-editing and experimental evolution, which are costly and time consuming, and require expertise in molecular biology and/or microbiology. We believe that the presented methodology demonstrates an innovative use of synthetic materials. Rather than trying to inhibit formation of biofilms, we have used synthetic polymers to induce the formation of these communities of bacteria, and exploited these polymer-induced biofilms for a beneficial application, in this case biocatalysis.

## 1. Introduction

The vast majority of bacteria live in biofilms, microbial communities where cells stick to each other, and are protected by an extracellular matrix of biopolymers.^1–7^ Biofilms are far more resistant than planktonic bacteria to environmental conditions, including extreme pH and temperatures, or to the presence of detrimental chemicals and metabolites, including antibiotic treatment.^2,4,8,9^ As a consequence, bacterial biofilms are often seen as problematic and difficult to eradicate, and have been associated for example with biofouling^10^ or hospital-acquired infections.^11^ This awareness of problematic biofilms means that the role of beneficial biofilms and microbial communities are often ignored and overlooked. For instance, biofilms of beneficial microorganisms are critical in maintaining a healthy microbiota, which plays a critical role in human, animal and plant health.^12–14^ As such, strategies to establish and maintain healthy microbiotas will have an impact in agriculture^15^ and consumer health,^16,17^ and have been at the heart of traditional manufacturing of foods such as cheese, vinegar or fortified wines.^18–20^ Moreover, the intrinsic resilience of biofilms can be exploited to underpin other beneficial applications, including bioremediation^21,22^ or biocatalysis.^4,23–26^

One of the challenges in exploiting beneficial biofilms for biotechnology or health is that some of the candidate microorganisms, such as probiotics^27,28^ or non-pathogenic strains of *Escherichia coli*,^29^ are not necessarily good at forming biofilms. Strains that form biofilms efficiently can appear as a result of evolution (*e.g.* the *E. coli* PHL644 strain used in this work),^30^ a process that often requires lengthy incubation times and multiple rounds of culture, something that limits its practicality. Alternatively, relevant genes for adhesion and biofilm formation can be introduced *via* genetic modification or synthetic biology approaches, although this process is often reserved to introduce genes needed for biocatalytic activity and bioprocessing. Moreover, for some applications such as bioremediation, food manufacture, or agriculture, the release of genetically modified organisms into the environment is a concern.

Interestingly, the switch between planktonic and biofilm lifestyles is often dictated by environmental factors such as the presence or absence of certain nutrients, pH, or temperature.^2^ Often, adhesion to surfaces and hosts can trigger this transition to a biofilm phenotype.^2^ We postulate here that a similar transition should be observed following the binding of microorganisms to synthetic polymers. We had already identified that this was the case for human pathogen *Vibrio cholerae*, that upon interaction with cationic polymers, would adopt a non-virulent sessile lifestyle, characterized by an increase in biofilm formation and a decrease in toxin production.^31,32^

**Scheme 1:**
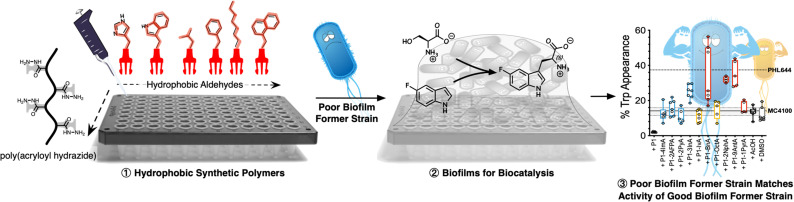
Schematic representation of experimental approach to polymer-induced biofilms for biocatalysis: hydrophobic polymers are prepared using an *in situ* screening strategy based on poly(acryloyl hydrazide) (step ①). Then, a poor biofilm former strain (MC4100) is incubated in the presence of these polymers to yield biofilms (step ②). This way, the poor biofilm former strain is able to match the performance of a good biofilm former strain (PHL644) in the biotransformation of serine and 5-fluoroindole into 5-fluorotryptophan (step ③).

In this manuscript, we show that synthetic polymers induce the nucleation of biofilms in biotechnology relevant *E. coli*, resulting in increased biocatalytic activity in a model biotransformation (Scheme 1). First, using a post-polymerization functionalization strategy, we prepare a set of linear polymers carrying mildly cationic, aromatic, heteroaromatic or aliphatic moieties. Then, using *E. coli* MC4100 as a model microorganism, we demonstrate that incubation with these synthetic polymers promotes clustering and the formation of microbial biofilms. This polymer-induced formation of biofilms was characterized by increased levels of biomass and curli (protein fibres on the bacterial surface responsible for adhesion to surfaces and thus biofilm formation), both key biofilm biomarkers in this organism. Our results indicate that hydrophobic polymers outperform mildly cationic polymers, with increasingly hydrophobic materials often resulting in higher levels of biofilm formation. Throughout the manuscript we demonstrate that, in the presence of these synthetic polymers, *E. coli* MC4100, a poor biofilm former, can reach levels of biomass production, and curli expression similar or higher than PHL644, a good biofilm former with a single point mutation in the OmpR regulator which increases curli expression. Finally, we show that these polymers can be used to increase the biocatalytic activity of engineered *E. coli* biofilms in the transformation of serine and 5-fluoroindole into 5-fluorotryptophan. All together, our results demonstrate that the use of these synthetic polymers as additives in microbial culture can be a cheap alternative to engineering microbial biofilms using gene editing or experimental evolution.

## 2. Results & discussion

### 2.1. Polymer-induced biofilm formation: screening chemical space

**Scheme 2:**
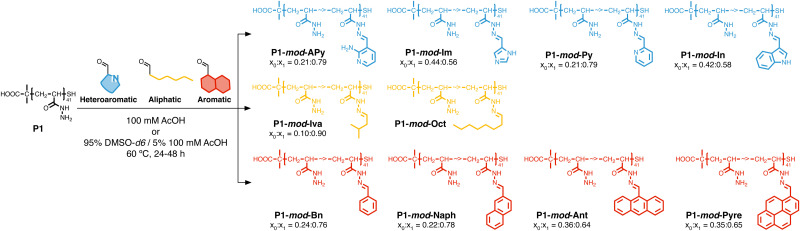
Synthesis of functional polymers P1-*mod*-aldehyde, and list of polymers prepared with aldehyde conversions.

Following our previous work with *V. cholerae*,^31,32^ for which we had seen an increase in biofilm formation upon incubation with cationic polymers, we postulate here that this polymer-induced biofilm formation can be exploited in other microorganisms for the production of biofilms for biotechnology. As mentioned above, the relevance of microbial biofilms for biotechnology is slowly becoming apparent, as the protective environment offered by biofilms can be exploited for food production,^18–20^ bioremediation^21,22^ or biocatalysis.^4,23–26^ In our case, we focused on biocatalysis, where biofilms can protect microbial cells from harsh conditions such as extreme pH or temperature, or from the presence of detrimental chemicals such as organic solvents. In our previous work, we had exploited cationic polymers to induce clustering in a range of bacteria, including *Vibrio harveyi*,^33–36^*V. cholerae*,^31,32^*Pseudomonas aeruginosa*,^34,35^*Staphylococcus aureus*^35^ and *E. coli*,^34,35^ and, as just mentioned, we observed increased biofilm formation for *V. cholerae*. However, we also observed that cationic polymers were toxic to some of the *E. coli* strains employed.^34^ Thus, we decided to explore mildly cationic polymers that would be partially protonated in the culture media. Since both electrostatic and hydrophobic interactions are involved in the initial stages of adhesion by bacteria to surfaces and hosts,^37^ we decided to also investigate hydrophobic polymers. To have quick access to this broad chemical space, we employed a post-polymerization modification strategy developed in our team (Scheme 2).^38^ This strategy relies on the functionalization of poly(acryloyl hydrazide) P1 with aldehydes under aqueous conditions, and the *in situ* evaluation of activity of the formed functional polymers. The post-polymerization modification is often done under mildly acidic conditions (here 100 mM acetic acid at pH 3), giving us versatility when exploring a broad chemical space, and access to functional polymers simply by choosing the required aldehydes. To date this *in situ* screening methodology has only been applied to the delivery of nucleic acids,^39–41^ and we wanted to demonstrate here its versatility to develop other functional polymers with biological relevance.

**Fig. 1:**
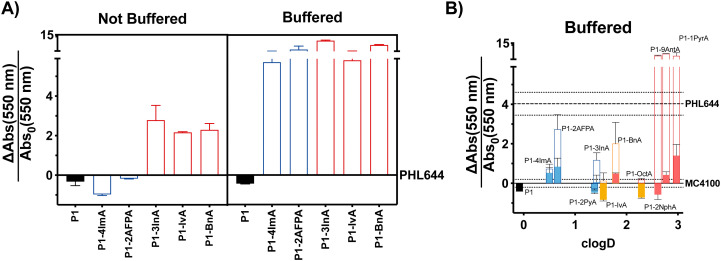
Biofilm formation as measured by crystal violet staining: fractional change in absorbance at 550 nm for *E. coli* PHL644 cultures (A) and *E. coli* MC4100 cultures (B) following incubation over 24 h in the presence of 0.05 mg mL^−1^ of P1 (black solid bar), aldehydes (solid coloured bars) and functional polymers P1-*mod*-aldehyde (hollow coloured bars). Data has been normalised and represents the fractional change in absorbance at 550 nm when compared to *E. coli* MC4100 cultures incubated in the absence of polymers (solid line). Fractional change in absorbance at 550 nm for *E. coli* PHL644 cultures incubated in the absence of polymers when compared to *E. coli* MC4100 cultures incubated in the absence of polymers is also shown for comparison (dashed line). Not buffered indicates incubation in 100 mM aqueous NaCl. Buffered indicates incubation in 100 mM phosphate buffer at pH 7. Means ± range from at least three biological replicates are shown. Full details of polymer *c*log *D* calculations are available in the ESI.†

With these considerations in mind, we decided to perform a first screening of the ability of synthetic polymers to induce biofilms in *E. coli* using isovaleraldehyde (IvA, aliphatic), benzaldehyde (BnA, aromatic) and 1*H*-indole-3-carbaldehyde (3InA, heteroaromatic) as representative hydrophobic aldehydes (Fig. 1A, red bars), and 1*H*-imidazole-4-carbaldehyde (4ImA, *cpK*_*aH*_ 5.4) and 2-amino-3-formylpyridine (2AFPA, *cpK*_*aH*_ 5.5) as representative mild cationic aldehydes (Fig. 1A, blue bars). Since the parent poly(acryloyl hydrazide) (P1, *cpK*_*aH*_ 3.3) should be partially protonated at pH 3, it was also investigated. *E. coli* K-12 strain PHL644 was used as a model strain,^30^ as it has already been investigated as a platform for biocatalysis using biofilms.^42–44^ Cationic polymers were prepared by mixing stock solutions of P1 and aldehyde in 100 mM acetic acid to give a final 125 mM concentration of aldehyde and a 1 : 1 ratio between aldehyde and hydrazide moieties. Hydrophobic polymers were prepared following the same protocol but using 95% DMSO-d6/5% 100 mM acetic acid as the solvent. Following incubation of P1 and the aldehydes over 24 h at 60 °C, aldehyde loading on the polymer was analysed by NMR as reported.^38,39^ In all cases this loading was consistent with our previous results, with most aldehydes giving approximately 70% loading (Table S1, ESI†), while bulky aldehydes such as 3InA gave slightly lower values (*i.e.* 58%). The formed polymers were then added without further purification to suspensions of *E. coli* PHL644 bacteria in a 100 mM aqueous solution of NaCl, to ensure that the polymers remained protonated after their preparation at pH 3. Following incubation for 24 h (Fig. 1A), 3 days (Fig. S7, ESI†) or 5 days (Fig. S8, ESI†), cultures were stained with crystal violet (CV) to monitor the amount of biomass produced as a metric for biofilm formation.^45^ Interestingly, under these conditions, only hydrophobic polymers were able to significantly increase the levels of crystal violet staining above those of the untreated bacteria, with cationic polymers mainly reducing the amount of staining (Fig. 1A and Fig. S7, S8, not buffered, ESI†). Seeing how hydrophobicity seemed to be the dominating effect, we decided to repeat the experiment, but using 100 mM phosphate buffer at pH 7 instead of the NaCl solution. This way, cationic moieties would be mainly neutralized, rendering all polymers hydrophobic. As expected, all polymers were able now to increase biofilm formation as measured by crystal violet staining (Fig. 1A and Fig. S7, S8, buffered, ESI†). P1 did not induce biofilm formation under these conditions, as it is probably still very hydrophilic, despite being less protonated.

### 2.2. Biofilm formation: hydrophobic polymers

Having identified that hydrophobicity was the main driving force for the increase in crystal violet staining, we decided to expand the range of hydrophobic aldehydes evaluated. Since both benzaldehyde (BnA) and 1*H*-indole-3-carbaldehyde (3InA) were the aldehydes that resulted in higher levels of crystal violet staining, we decided to investigate 2-naphthaldehyde (2Nph), anthracene-9-carbaldehyde (9AntA) and pyrene-1-carbaldehyde (1PyrA) as additional aromatic aldehydes (Fig. 1B, hollow red bars), and 2-pyridinecarboxaldehyde (2PyA) as an additional heteroaromatic (Fig. 1B, hollow blue bars), giving a total of 4 of each type of aldehyde. Only octylaldehyde (OctA) was investigated as an additional aliphatic aldehyde (Fig. 1B, hollow yellow bars), as the polymer derived from IvA gave the worst results in our previous experiment, specially after 3 (Fig. S7, ESI†) and 5 days of incubation (Fig. S8, ESI†). *E. coli* K-12 strain MC4100 was investigated this time, as it is a poor biofilm former lacking the mutation in OmpR that confers increased curli expression and biofilm formation to strain PHL644. At this stage, we wanted to evaluate if the polymers were able to increase biofilm formation also for strains that are not as efficient in establishing biofilms. Although we didn’t observe a clear correlation between the amount of biomass produced and the hydrophobicity of the polymers (quantified here by *c*Log *D*, see ESI† for details), it was clear at this stage that, much like for *E. coli* PHL644, hydrophobic polymers also increased the amounts of biofilm formed by MC4100 (Fig. 1B and Fig. S9, ESI†). Overall, polymers derived from aromatic aldehydes gave the best results. We wanted to ensure that the effect was not due to the presence of excess of unreacted aldehyde, or to the addition of traces of acetic acid and DMSO used in the preparation of the functional polymers. To our delight, none of the aldehydes induced significant levels of crystal violet staining, with only 4ImA giving similar levels of staining to its functionalized polymer P1-*mod*-4ImA (Fig. 1B, solid coloured bars). Similarly, incubation with the buffers used for polymer preparation resulted in little to no increase in staining with crystal violet (Fig. S10, ESI†), suggesting that overall the functional polymers were the agents responsible for the enhancement in biofilm formation.

We then wanted to compare the amount of biomass produced by MC4100 in the presence of these polymers with that of PHL644, which is a good biofilm former. As expected, in the absence of polymers, PHL644 is able to produce, after 24 h, approximately 4 times more biomass (as measured by CV staining) than MC4100 (Fig. 1B, dashed line), and twice the amount of biomass after 48 h (Fig. S9, dashed line, ESI†). Interestingly, in the presence of the aromatic polymers, MC4100 was able to produce comparable, if not higher levels of biomass. This increase was particularly notable for the most hydrophobic aromatic polymers P1-*mod*-2NphA, P1-*mod*-9AntA and P1-*mod*-1PyrA, which after 24 h helped MC4100 reach significantly higher levels of biomass production than PHL644 (Fig. 1B, red hollow bars). This increase in biomass production for MC4100 in the presence of these synthetic polymers is remarkable, as a single point mutation is responsible for the increased ability of PHL644 to form biofilms,^30^ suggesting that, under the right conditions, incubation with synthetic polymers can result in phenotypic changes similar to those obtained using genetic engineering.

### 2.3. Aggregation of bacteria

**Fig. 2:**
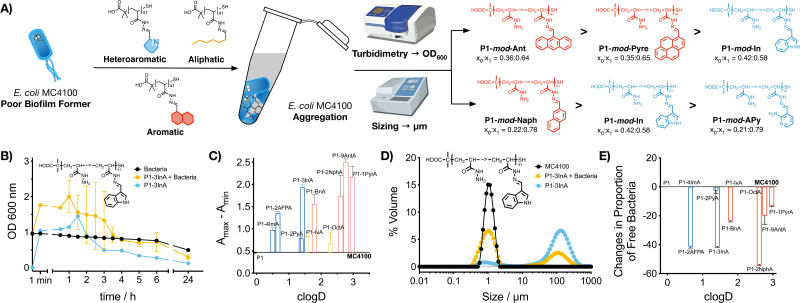
Aggregation of bacteria: (A) schematic representation of polymer-induced aggregation of bacteria, the techniques used for its characterization and the best preforming polymers identified with each technique. (B) Optical density at 600 nm for *E. coli* MC4100 cultures following incubation for 24 h in the absence (black) and presence of 0.5 mg mL^−1^ of P1-*mod*-3InA (yellow). Optical density at 600 nm of 0.05 mg mL^−1^ of P1-*mod*-2InA suspended in culture media (blue) shown for comparison. (C) Changes to optical density at 600 nm for *E. coli* MC4100 cultures in the presence of 0.5 mg mL^−1^ of functional polymers P1-*mod*-aldehyde. (D) Size distribution of suspensions of MC4100 cultures (black) and *E. coli* MC4100 cultures in the presence of 0.05 mg mL^−1^ of P1-*mod*-3InA (yellow), following incubation over 48 h. Size distribution of suspensions of P1-*mod*-3InA in culture media (blue) shown for comparison. (E) Changes in the proportion of free bacteria in suspensions of *E. coli* MC4100 incubated in the presence of 0.05 mg mL^−1^ of functional polymers P1-*mod*-aldehyde for 48 h. Data has been normalised and represents the difference when compared to *E. coli* MC4100 cultures in the absence of polymers. Means ± SD from at least two biological replicates are shown.

Our previous work with cationic polymers indicates that changes in microbial physiology, including biofilm formation, are the result of polymer-induced clustering of bacteria.^31–36^ Here, we wanted to evaluate if a similar aggregation was being induced by hydrophobic polymers. To this end, aggregation of bacteria was first evaluated monitoring changes in the optical density for *E. coli* MC4100 cultures in the absence and presence of these functional polymers (Fig. 2B and C, P1-*mod*-3InA shown as representative example, full data in Fig. S25–S35, ESI†). In this assay, bacteria are resuspended at high cell density, and allowed to settle. In the absence of clustering (Fig. 2B, black trace) *E. coli* MC4100 slowly sediments, resulting in a gradual decrease in optical density at 600 nm. When bacteria aggregate to form clusters, two phenomena can be observed. On one hand, aggregation can lead to an increase in optical density (Fig. 2B, yellow trace, P1-*mod*-3InA shown as representative example), as a result of bacterial clusters scattering more light than individual bacteria. These aggregates will gradually sediment, leading to a decrease in optical density. On the other hand, fast sedimentation can already happen at early timepoints in the experiments, when aggregation of bacteria is very fast.^33,46^ The timing of these two phenomena is highly dependent on small changes in the concentration of bacteria and polymer and, as such, there is a lot of batch-to-batch variability. In our case, all functional polymers induced changes in the optical density of the culture when compared to untreated bacteria. These changes were more obvious when the difference between the maximum and minimum optical density was plotted (Fig. 2C). Once again, aromatic polymers seemed to be the best performers including heteroaromatic P1-*mod*-2AFPA and P1-*mod*-3InA. Like for biofilm formation, most of the aldehydes did not induce any changes in the optical density of the cultures (Fig. S25B–S31B, ESI†) except for very hydrophobic aldehydes 2NphA (Fig. S33B, ESI†), 9AntA (Fig. S34B, ESI†) and 1PyrA (Fig. S35B, ESI†) and to a certain extent OctA (Fig. S32B, ESI†). However, in all cases, the changes in optical density observed for aldehydes were different from those observed in the presence of the polymers and, overall, suggest that the main driving force for the aggregation of bacteria is the presence of these hydrophobic polymers.

Interestingly, when we evaluated the effect of the polymers on the optical density of the culture media, most functional polymers showed similar changes to the optical density than those in the presence of bacteria (Fig. 2B and Fig. S25A–S35A, blue trace, ESI†). These changes in optical density in the absence of bacteria suggest that the functional polymers are not soluble in this culture media, and therefore precipitate out of solution. This effect is also observed for the most hydrophobic aldehydes (Fig. S32–S35, ESI†) that quickly precipitate when suspended in culture media, while the least hydrophobic aldehydes did not induce any changes in the optical density of the culture media (Fig. S26–S31, ESI†).

To further characterize polymer-induced aggregation, we decided to measure the size of the aggregates formed when polymers were suspended in media using light scattering, both in the presence and absence of bacteria (Fig. 2D, P1-*mod*-3InA shown as representative example, full data in Fig. S36–S44, ESI†). This technique was able to confirm that these polymers were insoluble in the growth media and aggregates could be observed, in particular for very hydrophobic compounds. More importantly, the polymers were also able to reduce the concentration of free bacteria in suspension (Fig. 2E) with 2AFPA, 3InA and 2NphA leading to approximately a 40% reduction. However, with this experiment we were unable to determine whether the polymers were interacting directly with bacteria and leading to aggregation. Regardless, when this data was compared to that obtained from the crystal violet staining (Fig. 1), we could see similar trends, with polymers derived from aromatic aldehydes, and 2AFPA and 3InA inducing the biggest changes. Overall we think that, independently of their precise mode of action, these polymers are behind the increase in biofilm formation observed.

### 2.4. Polymer-induced biofilm formation: curli expression

**Fig. 3:**
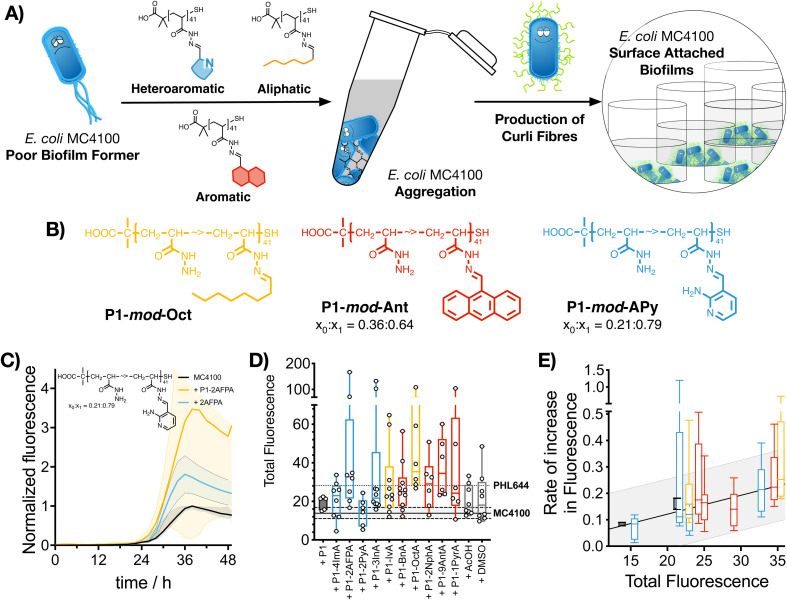
Curli expression measured using a GFP reporter strain: (A) Schematic representation of the formation of curli fibres following polymer-induced aggregation of bacteria, which leads to surface-attached biofilms. (B) Best preforming polymers identified in this assay. (C) Green fluorescence against time for *E. coli* MC4100 pJLC-T cultures following incubation in 100 mM phosphate buffer at pH 7 in the absence (black, *n* = 10) and presence of 0.05 mg mL^−1^ of P1-*mod*-2AFPA (yellow, *n* = 8) or 2AFPA (blue, *n* = 3). Mean ± 95% confidence intervals are shown. (D) Total GFP fluorescence for *E. coli* MC4100 pJLC-T cultures in the presence of 0.05 mg mL^−1^ of P1 (black solid box) and functional polymers P1-*mod*-aldehyde (hollow boxes). (E) Rate of increase of green fluorescence for *E. coli* MC4100 pJLC-T cultures in the absence and presence of 0.05 mg mL^−1^ of functional polymers P1-*mod*-aldehyde. For box and whisker plots, median is shown as a line. Box extends from 25th to 75th percentile while whiskers go from minimum to maximum value. Fit to a straight line together with prediction bands are shown.

Having identified that hydrophobic polymers were promoting aggregation in *E. coli* and an increase in crystal violet staining, we wanted to evaluate next if bacteria were producing some of the key biofilm biomarkers. The primary stage of *E. coli* biofilm formation is the adhesion of cells to a solid surface *via* the adhesin curli, an amyloid fibre that projects from the cell surface and binds to both biotic and abiotic surfaces.^47^ The expression of curli is mediated by two operons, *csgBA* and *csgDEFG*. Since the *csgBA* operon encodes the main structural subunit protein of curli CsgA, and the nucleator protein CsgB, a reporter plasmid (pJLC-T) was used to measure *csgB* promoter activity.^48^ The *csgB* promoter region was fused to *gfp*, the gene encoding green fluorescent protein (GFP) to monitor expression of curli through time using a simple fluorescence readout. Curli expression is maximal at temperatures of around 30 °C and in conditions of low osmolarity.^47^ Under these conditions, and in the absence of polymers, it took *E. coli* MC4100 more than 20 h to produce significant levels of GFP fluorescence, reaching maximum GFP expression approximately after 38 h of incubation (Fig. 3C, black trace). When incubated in the presence of functional polymers, no significant changes in the onset of GFP fluorescence were observed (Fig. S24, ESI†). However, the total amount of fluorescence was significantly affected in the presence of polymers (Fig. 3C, yellow trace, P1-*mod*-2AFPA shown as representative example, full data in Fig. S11–S23, ESI†). As before, hydrophobicity seemed to be a significant factor and, polymers derived from aromatic aldehydes and 2AFPA gave the strongest responses (Fig. 3D). This increase in total fluorescence seemed to be the result of an increase in the rate of activation of the *csgB* promoter (Fig. 3E). Interestingly, both polymers derived from aliphatic aldehydes, P1-*mod*-IvA and P1-*mod*-OctA, were also inducing significant levels of GFP fluorescence (Fig. 3B, yellow bars) despite being one of the weakest performers in the crystal violet and aggregation assays before. However, these aliphatic polymers couldn’t induce clustering (Fig. 2C and E, yellow bars) or an increase in biomass (Fig. 1B, yellow bars), suggesting that in the presence of these aliphatic polymers, MC4100 was unable to establish surface-attached biofilms. This observation is in agreement with some recent results that show that curli expression in *E. coli* K-12 strains is upregulated in planktonic cells and floating biofilms, also known as pellicles, but gets downregulated once bacteria settle on solid surfaces.^49^

When we compared curli expression for MC4100 in the presence of these polymers to that of PHL644, we could see that, like in the case of biomass, MC4100 was now reaching levels of GFP fluorescence similar to, if not higher than, those for PHL644 (Fig. 3D). This increase in fluorescence was particularly the case for P1-*mod*-2AFPA, P1-*mod*-OctA and P1-*mod*-9AntA, the best performing polymers in this assay. As before, incubation with aldehydes resulted in moderate increases in GFP fluorescence but, in most cases, these increase was lower than that of the corresponding functional polymers (Fig. S11–S23, ESI†). Interestingly, none of the polymers was able to induce curli expression in MC4100 faster than PHL644 (Fig. S24, ESI†), for which significant levels of GFP fluorescence could be observed as early as 10 h after incubation. This difference suggests that a different mechanism of biofilm enhancement occurs for PHL644 *versus* our polymers. In PHL644 the mutation in *ompR* activates *csgB* expression, *via* the regulator CsgD, in both exponential and stationary phase, although more in stationary phase.^50^ Alternatively, we propose here that our polymers mediate clustering which then triggers a physiological response leading to curli production, taking a little longer than the *ompR234* mutation. These results again suggest that, while the polymers may not be able to mimic all the changes introduced at the genetic level by experimental evolution and gene editing, they can be useful tools to modulate key phenotypes associated with biofilm formation, such as biomass and curli expression.

### 2.5. Biocatalysis: synthesis of 5-fluorotryptophan

**Fig. 4:**
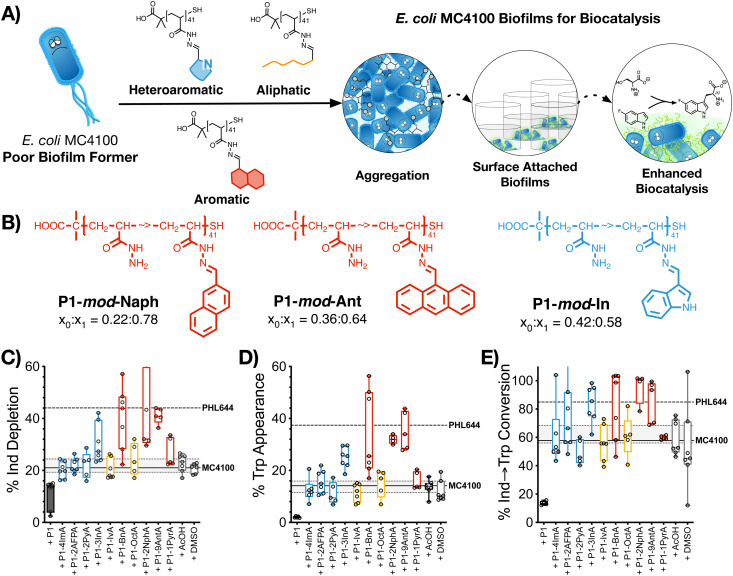
Biocatalytic activity: (A) schematic representation of polymer-induced biofilms for biocatalysis. (B) Best preforming polymers identified in this assay. (C) Percentage of 5-fluoroindole depletion, (D) 5-fluorotryptophan appearance and (E) conversion of 5-fluoroindole to 5-fluorotryptophan for *E. coli* MC4100 pSTB7 cultures following 48 h of incubation in 100 mM phosphate buffer at pH 7 in the presence of 0.05 mg mL^−1^ of P1 (black solid box) and functional polymers P1-*mod*-aldehyde (hollow boxes), followed by incubation with reaction buffer for another 24 h. Median is shown as a line. Box extends from 25th to 75th percentile while whiskers go from minimum to maximum value. Values for *E. coli* MC4100 (solid line with 25th to 75th percentile) and *E. coli* PHL644 cultures (dashed line) incubated in the absence of polymers shown for comparison.

The last stage of our work was to evaluate if these hydrophobic polymers could help a poor biofilm former such as MC4100 establish biofilms for biocatalysis. Previous work from our team had suggested that this strain is significantly less efficient than PHL644 in the synthesis of halotryptophans from serine and haloindoles.^43^ Here, we decided to use the biotransformation of serine and 5-fluoroindole into 5-fluorotryptophan as a model reaction. To this end, MC4100, transformed with pSTB7 (expressing the *Salmonella* typhimurium tryptophan synthase which catalyses this biotransformation),^51^ was incubated in the absence and presence of the functional polymers as described above, and then treated with a buffer containing both starting materials for this biotransformation. The supernatants were then collected and the amount of 5-fluoroindole depleted (Fig. 4C), and 5-fluorotryptophan produced (Fig. 4D), quantified using HPLC (Fig. S47–S57, ESI†). It should be noted that this reaction is reversible due to the degradation of 5-fluorotryptophan catalysed by the native *E. coli* enzyme tryptophanase, TnaA.^52^ Thus, to quantify the efficiency of this biotransformation, an additional parameter, percentage conversion of 5-fluoroindole to 5-fluorotryptophan, was calculated (Fig. 4E). In the absence of functional polymers, MC4100 pSTB7 was only able to consume 21% of 5-fluoroindole (Fig. 4C) to yield 14% of 5-fluorotryptophan, in line with what has been reported for this strain.^43^ As expected, MC4100 pSTB7 was less active than PHL644 pSTB7, which was able to consume 44% of 5-fluoroindole while producing 37% of 5-fluorotryptophan (Fig. 4D). This increased efficiency was reflected in the selectivity of the biotransformation, with PHL644 pSTB7 outperforming MC4100 pSTB7 by 85% *vs.* 58% (Fig. 4E).

In line with our previous results, when MC4100 pSTB7 was incubated with our hydrophobic polymers we observed an increase in both the amount of 5-fluoroindole consumed and 5-fluorotryptophan produced by this strain, with aromatic P1-*mod*-BnA, P1-*mod*-2NphA and P1-*mod*-9AntA being again the best performing polymers. While none of the polymers were able to help MC4100 outperform PHL644 in these two areas, incubation of MC4100 pSTB7 with P1-*mod*-BnA resulted in the same level of 5-fluoroindole depletion (Fig. 4C) while incubation with P1-*mod*-2NphA and P1-*mod*-9AntA gave similar levels of 5-fluorotryptophan to those produced by PHL644 pSTB7 (Fig. 4D). More importantly, when we analysed the efficiency of the transformation of 5-fluoroindole to 5-fluorotryptophan (Fig. 4E), these three aromatic polymers were helping MC4100 pSTB7 outperform PHL644 pSTB7 and even heteroaromatic P1-*mod*-3InA was now reaching similar levels of conversion.

**Fig. 5:**
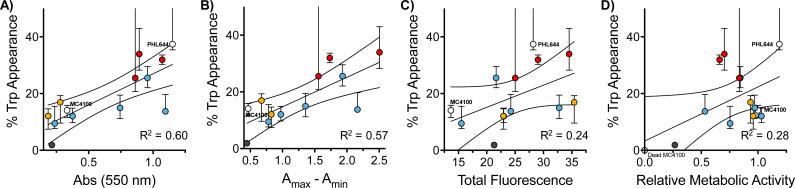
Correlation of relevant phenotypes with 5-fluorotryptophan production: percentage of 5-fluorotryptophan appearance as a function of biomass (as measured by crystal violet staining) (A), clustering (as measured by changes to optical density at 600 nm) (B), curli production (as measured by total green fluorescence) (C), and relative metabolic activity (as measured by resorufin fluorescence) (D), for *E. coli* MC4100 cultures following 48 h of incubation in 100 mM phosphate buffer at pH 7 in the absence (lower white dot) and presence of 0.05 mg mL^−1^ of P1 (black solid dot) and functional polymers P1-*mod*-aldehyde (coloured dots), followed by incubation with reaction buffer for another 24 h. Where relevant, data for *E. coli* PHL644 cultures is shown (top white dot). Median and 25th to 75th percentiles shown for at least 4 replicates.

Interestingly, when we evaluated how this improvement in MC4100 pSTB7's ability to carry out this biotransformation correlated with some of the phenotypes previously investigated, we observed that this increase in performance was better correlated to the ability of these polymers to increase the amount of biomass and promote aggregation in MC4100 pSTB7 (Fig. 5A and B) but not to curli expression (Fig. 5C). This correlation is in line with our observation that this adhesin is overexpressed in floating but not attached biofilms,^49^ and that biofilms are a suitable platform for this biotransformation.^42–44^ Similarly, there was a very poor correlation to the degree of functionalization (Fig. S58, ESI†) or to the effect these polymers have on the metabolic activity of MC4100 (Fig. 5D). Here, we relied on the reduction of resazurin to highly fluorescent resorufin by healthy cells, an assay that has been often used to monitor aerobic respiration.^53^ While most functional polymers reduced the metabolic activity of MC4100, this organism remained viable in all cases, as shown by its ability to produce curli or carry out biocatalysis in the presence of these polymers. Only incubation in the presence of the unfunctionalized polymer P1 (Fig. 5D, black dot) yielded levels of resorufin fluorescence close to those produced by dead cells; P1 was also one of the worst performing polymers in the functional assays above, in particular in terms of biomass production (Fig. 1) and biocatalytic activity (Fig. 4). It is worth noting that PHL644 showed a higher metabolic activity than MC4100, something that may support its increased ability to establish biofilms and perform biocatalysis. It is remarkable thus that, while incubation with functional polymers reduced the metabolic activity of MC4100 and, that in all cases, the metabolic activity of this strain was lower than PHL644 (Fig. 5D), the efficiencies of the biotransformations were reaching levels similar to those of PHL64.

## 3. Conclusion

In conclusion, we have presented here a new methodology to induce biofilms for biocatalysis in *E. coli*, one of the workhorses in biotechnology. In our approach, synthetic polymers are used as simple additives during microbial culture, promoting cell clustering and the formation of biofilms. First, using an *in situ* post-polymerization modification methodology, we identified that hydrophobic polymers outperformed mildly cationic polymers in promoting biofilms for PHL644, an *E. coli* strain that forms biofilms well. Then, we demonstrate that these hydrophobic polymers induced biofilm formation also in MC4100, an isogenic strain that forms biofilms poorly. Using this *in situ* screening methodology we identified which chemistries are best suited for this application, with aromatic and heteroaromatic polymers outperforming the equivalent aliphatic derivatives. Our results indicate that these synthetic polymers promote cell clustering, resulting in an increase in biomass and curli production, both key phenotypes in establishing *E. coli* biofilms. Finally, we demonstrate that the biocatalytic activity of *E. coli* MC4100 in a model transformation increased in the presence of selected polymers. All together, our results indicate that incubation with these synthetic polymers helps MC4100 match and even outperform PHL644 in the assays investigated, including the biocatalytic transformation employed. Since PHL644 carries a single point mutation responsible for its increased ability to colonise surfaces and form biofilms, our results suggest that incubation with polymers can induce similar phenotypic changes than those observed following gene editing.

We believe the presented work significantly advances the state-of-the art, providing a new methodology to induce biofilms for biocatalysis. The simplicity of this methodology, coupled with its versatility, has allowed us to identify the best performing chemistries and a set of hydrophobic polymers that increases the biocatalytic activity of *E. coli*. Moreover, we believe this work has an impact beyond biocatalysis, and should be of relevance to others investigating beneficial applications of biofilms. A similar strategy could be employed to induce biofilms in other microorganisms such as probiotics or yeasts, and develop new applications in food science, agriculture, bioremediation or health. Our ambition is that this methodology can be used both by experts and non-experts to develop new applications in these fields. Our efforts to identify new applications of the reported *in situ* screening methodology, new polymer compositions and topologies that promote biofilms in other beneficial microorganisms, and how these microorganisms are regulating the phenotypes observed at the genetic level, will be reported in due course.

## Author contributions

P. A., T. W. O. and P. F.-T. conceived and designed the experiments. P. F.-T. and T. W. O. secured funding. P. A. and A. R. prepared P1, and P. A. performed all other experiments. P. A., T. W. O. and P. F.-T. analyzed the data and wrote the paper, with all other authors contributing to the final version of the manuscript.

## Conflicts of interest

The authors declare the following competing financial interest: P. A., T. W. O. and P. F. -T. are named inventors on a patent application (WO 2021/209765 A2) related to this work.

## Supplementary Material

MH-009-D2MH00607C-s001
