# Supplementary material for: Polymer-induced biofilms for enhanced biocatalysis
Source: Mater Horiz. 2022 Jul 21;9(10):2592–602. doi: 10.1039/d2mh00607c (PMC9528183; doi:10.1039/d2mh00607c)
Supplement: MH-009-D2MH00607C-s001 [file MH-009-D2MH00607C-s001.pdf]

Electronic Supplementary Material (ESI) for Materials Horizons.
This journal is © The Royal Society of Chemistry 2022

Supporting Information

Polymer-Induced Biofilms for Enhanced Biocatalysis

Pavan Adoni,[1,2,3] Andrey Romanyuk,[1,3,] Tim W. Overton[2,3,*] and Paco Fernandez-Trillo.[1,3,4*].

[1] School of Chemistry, [2] School of Chemical Engineering and [3] Institute of Microbiology and Infection, University of Birmingham, Edgbaston, Birmingham, UK, B15 2TT [4] Departamento de Química, Facultade de Ciencias and Centro de Investigacións Científicas Avanzadas (CICA), Universidade da Coruña, 15071 A Coruña, Spain.

t.w.overton@bham.ac.uk, f.fernandez-trillo@bham.ac.uk

1. Table of Contents

1. Table of Contents 1
2. General materials and methods 2
3. Media and preparation of polymer-cell suspensions 2
4. Bacterial Strains and plasmids 2
5. Hydrophobic Polymer Synthesis and Characterization 3
5.1. Poly(acryloyl hydrazide) P1 3
5.2. Synthesis of hydrophobic polymers: Poly(acryloyl hydrazide)–aldehyde coupling 4
5.3. Calculation of degree of functionalization using ^{1}H-NMR 4
5.4. Calculation of water:Octanol partition coefficients at pH 7 (cLogD) 6
6. Biofilm formation 6
6.1. Biofilm quantification using crystal violet 6
6.2. Curli expression using GFP reporter 8
7. Aggregation of bacteria 13
7.1. Spectrophotometric cell clustering 13
7.2. Sizing of polymer-bacteria aggregates 17
8. Biocatalysis: Synthesis of 5-fluorotryptophan 21
9. Metabolic Activity: Reduction of resazurin to resofurin 25

2. General materials and methods

Nuclear Magnetic Resonance (NMR) spectra were recorded on either a Bruker Avance III 300 MHz or a Bruker Avance III 400 MHz spectrometer. Chemical shifts are reported in ppm (units) referenced to the following solvent signals: dimethylsulfoxide (DMSO)-*d6* H 2.50 and D2O H 4.79. Ultraviolet/visible (UV-vis) measurements were performed using a Cary 50 spectrophotometer. Fluorescence measurements/assays were performed using a CLARIOstar PLUS plate reader. Purification of polymers was performed by dialysis in deionised water at room temperature for a minimum of 48 h using a Spectra/Por 6 1000 Molecular weight cut-off (MWCO) 38 mm width membrane. Poly(acryloyl hydrazide) DP=40 **P1** was prepared and characterized as previously described.[2] Gel Permeation Chromatography (GPC) was performed with a Shimadzu Prominence LC-20A fitted with a Thermo Fisher Refractomax 521 Detector and a SPD20A UV-vis Detector. Dulbecco's Phosphate Buffered Saline (0.0095 M PO_4) without Ca and Mg was used as the eluent at a flow rate of 1 mL min^{-1}. The instrument was fitted with an Agilent PL aquagel-OH column (300 × 7.5 mm, 8 mm) and run at 35 °C. Molecular weights were calculated based on a standard calibration method using poly(ethylene oxide). All aldehydes were purchased from either Sigma-Aldrich®, Fisher Scientific®, VWR® or Acros®, and used without further purification. All solvents were Reagent grade or above, purchased from Sigma-Aldrich®, Fisher Scientific® or VWR®, and used without further purification. cpK_{aH} were calculated using MarvinSketch® Protonation plugin.

3. Media and preparation of polymer-cell suspensions

Escherichia coli was grown on Luria-Bertani-agar (LB) plates (10 g/L tryptone, 5 g/L yeast extract, 10 g/L NaCl, 15 g/L bacteriological agar) (Sigma, UK). Cultures were prepared by selecting a single colony from the plate of the required strain and grown overnight in 10 mL LB broth (5 g/L tryptone, 2.5 g/L yeast extract, 5 g/L NaCl (Sigma, UK). For experiments involving monitoring the expression of curli using the reporter plasmid pJLC-T, growth media was supplemented with 10 µg/mL tetracycline. For experiments involving biotransformations using the plasmid pSTB7, growth media was supplemented with 100 µg/mL ampicillin. Cells were re-inoculated to 1% in LB in the morning and grown for 3 h until an OD_{600} of 0.2 was reached. Cells were then centrifuged down (10 min, 3900 rpm) and washed twice with water (2 x 10 mL). The pellet was resuspended in 0.1 M KH_2PO_4/K_2HPO_4 so that an OD_{600} of 0.2 was reached. Polymers were then added to the culture at 0.1 mg/mL, and the culture supplemented with an equal volume of standard M63 minimal media (100 mM KH_2PO_4, 15 mM $(NH_4)_2SO_4$, 1 mM $MgSO_4$, 1.8 µM $FeSO_4$, 10 mM glucose, 0.5% thiamine, 40 µg/mL L-cysteine adjusted to pH 7 using KOH pellets). Hence final polymer concentration was 0.05 mg/mL in 1:1 (v/v) 0.1 M KH_2PO_4/K_2HPO_4 and 1X M63. This suspension was then incubated at 30 °C, 150 rpm for the required amount of time, after which analysis would be performed. Where appropriate, an identical control experiment was also run whereby the respective aldehyde (rather than the polymer-aldehyde conjugate) was added to the cells at the same concentration. Polymer control experiments were also performed without the presence of bacteria.

4. Bacterial Strains and plasmids

Two *E. coli* K-12 strains MC4100: *araD139 Δ(argF-lac)U169 rpsL150 relA1 flbB5301 deoC1 ptsF25 rbsR* and PHL644: MC4100 *malA-kan ompR234* were used in this study.[3] Reporter plasmid pJLC-

---

[2] Crisan, D. N.; Creese, O.; Ball, R.; Brioso, J. L.; Martyn, B.; Montenegro, J.; Fernandez-Trillo, F. Poly(Acryloyl Hydrazide), a Versatile Scaffold for the Preparation of Functional Polymers: Synthesis and Post-Polymerisation Modification. *Polym. Chem.* **2017**, *8* (31), 4576–4584. https://doi.org/10.1039/C7PY00535K.

[3] Vidal, O.; Longin, R.; Prigent-Combaret, C.; Dorel, C.; Hooreman, M.; Lejeune, P. Isolation of an *Escherichia Coli* K-12 Mutant Strain Able To Form Biofilms on Inert Surfaces: Involvement of a New OmpR Allele That Increases Curli Expression. *J. Bacteriol.* **1998**, *180* (9), 2442–2449. https://doi.org/10.1128/jb.180.9.2442-2449.1998

T comprises the *E. coli* MC4100 *csgD-csgB* intergenic region upstream of the gene encoding eGFP with a C-terminal AANDEN-YALVA tag which reduces GFP half-life to around 60 min cloned into the EcoRI-HindIII sites of pPROBE'-TT upstream of the *gfp* gene.[4] pPROBE'-TT encodes tetracycline resistance and has a pBBR1 origin of replication.[5] pSTB7, a pBR322-based plasmid containing the *Salmonella enterica* serovar Typhimurium TB1533 *trpBA* genes and encoding ampicillin resistance,[6] was purchased from the American Type Culture Collection (ATCC 37845). *E. coli* strains were transformed with plasmids using the heat shock method.[4]

5. Hydrophobic Polymer Synthesis and Characterization

5.1. Poly(acryloyl hydrazide) P1

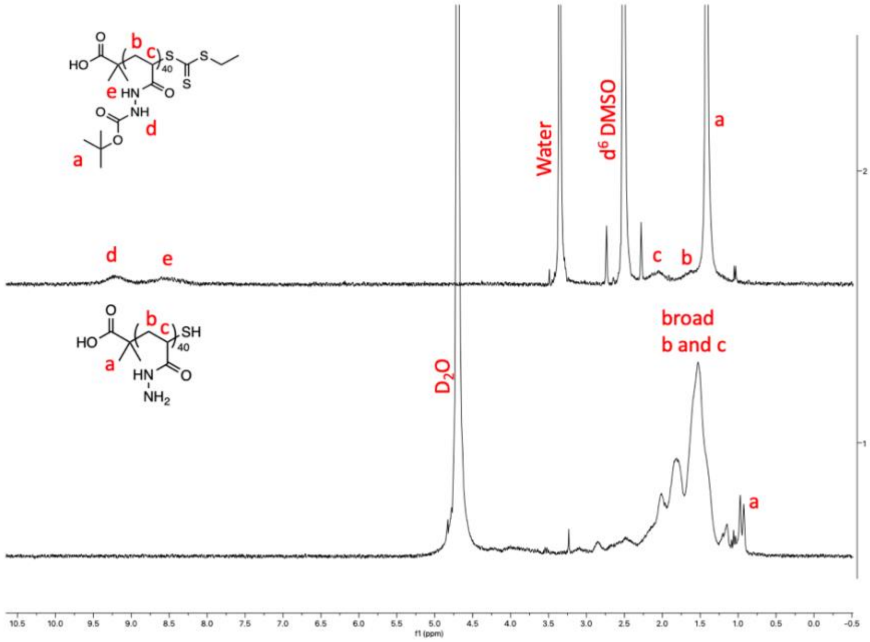

Figure S1 - poly(acryloyl hydrazide) P1 NMR: Top: ^{1}H-NMR of purified **boc-P1**, 1H NMR (300 MHz, DMSO-d6) δ (ppm) 8.85-9.53 (br, 1H, NH), 8.10-8.87 (br, 1H, NH), 1.90-2.23 (br, 1H, CH2CH), 1.64 (br, 11H, 9H in C(CH3)3, 2H in CHCH2), 1.02 (m, 6H, (C(CH3)2). Bottom: ^{1}H-NMR of **P1**. 1H NMR (300 MHz, D2O) δ (ppm) 1.27-2.30 (br, 3H), 0.97 (s, 3H), 0.92 (s, 3H).

[4] Leech, J.; Golub, S.; Allan, W.; Simmons, M. J. H.; Overton, T. W. Non-Pathogenic *Escherichia Coli* Biofilms: Effects of Growth Conditions and Surface Properties on Structure and Curli Gene Expression. *Arch. Microbiol.* **2020**, *202* (6), 1517–1527. https://doi.org/10.1007/s00203-020-01864-5.
[5] Miller, W. G.; Leveau, J. H. J.; Lindow, S. E. Improved Gfp and InaZ Broad-Host-Range Promoter-Probe Vectors. *Mol Plant-microbe Interactions* **2000**, 13 (11), 1243–1250. https://doi.org/10.1094/mpmi.2000.13.11.1243.
[6] Kawasaki, H.; Bauerle, R.; Zon, G.; Ahmed, S. A.; Miles, E. W. Site-Specific Mutagenesis of the Alpha Subunit of Tryptophan Synthase from *Salmonella* Typhimurium. Changing Arginine 179 to Leucine Alters the Reciprocal Transmission of Substrate-Induced Conformational Changes between the Alpha and Beta 2 Subunits. *J Biol Chem* **1987**, *262* (22), 10678–10683. https://doi.org/10.1016/s0021-9258(18)61017-8.

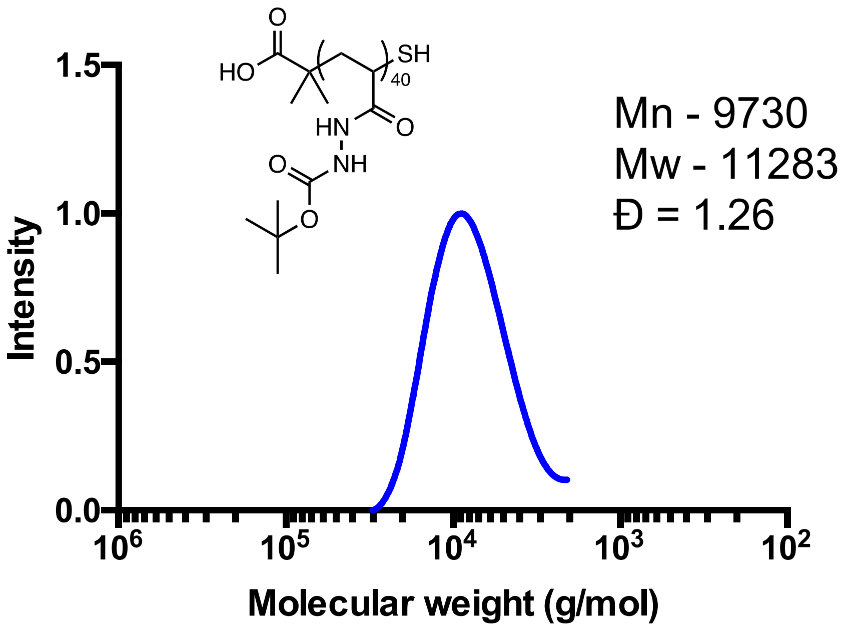

Figure S2 - poly(acryloyl hydrazide) P1 NMR: GPC chromatogram of **P1** (DP 40).

5.2. Synthesis of hydrophobic polymers: Poly(acryloyl hydrazide)–aldehyde coupling

Aldehydes were mixed with poly(acryloyl hydrazide) **P1** such that a 1:1 ratio of aldehyde:hydrazide groups was formed (not a 1:1 ratio of **P1**:aldehyde). Typically this protocol involved incubating **P1** (8.01 mg, 0.093 mmol hydrazides for a DP 40 polymer) with a molar equivalent of the required aldehyde to give a 125 mM solution in 95% DMSO-d6/5% 100 mM acetic acid for all aldehyde couplings except 2-amino-3-formylpyridine (**2AFPA**) and imidazole-4-carboxaldehyde (**4ImA**) which were reacted in 100 mM acetic acid. The mixture was incubated and stirred at 60 °C for 24-48 h to give the functional polymer which was then characterised by 1H-NMR as reported,[2] and used without further purification. **P1-*mod*-4ImA**, **P1-*mod*-3InA**, **P1-*mod*-IvA**, **P1-*mod*-OctA** and **P1-*mod*-2NphA** have been reported before.[2] **P1-*mod*-2AFPA**, **P1-*mod*-2PyA**, **P1-*mod*-9AntA** and **P1-*mod*-1PyrA** are new compounds and characterization details are given below (**Figure S3-Figure S6**).

5.3. Calculation of degree of functionalization using ^{1}H-NMR

Degree of functionalization for **P1-*mod*-aldehyde** was calculated from the ^{1}H-NMR spectra by comparing the integration of the residual aldehyde (between 9.5-11 ppm depending on the aldehyde, XH) against the overall number of protons in the aromatic and aldehyde region (between 5.5-11 ppm depending on the aldehyde, 1H from aldehyde or hydrazone, YH from the aromatic protons). Total number of protons in this region before and after functionalization remains constant.

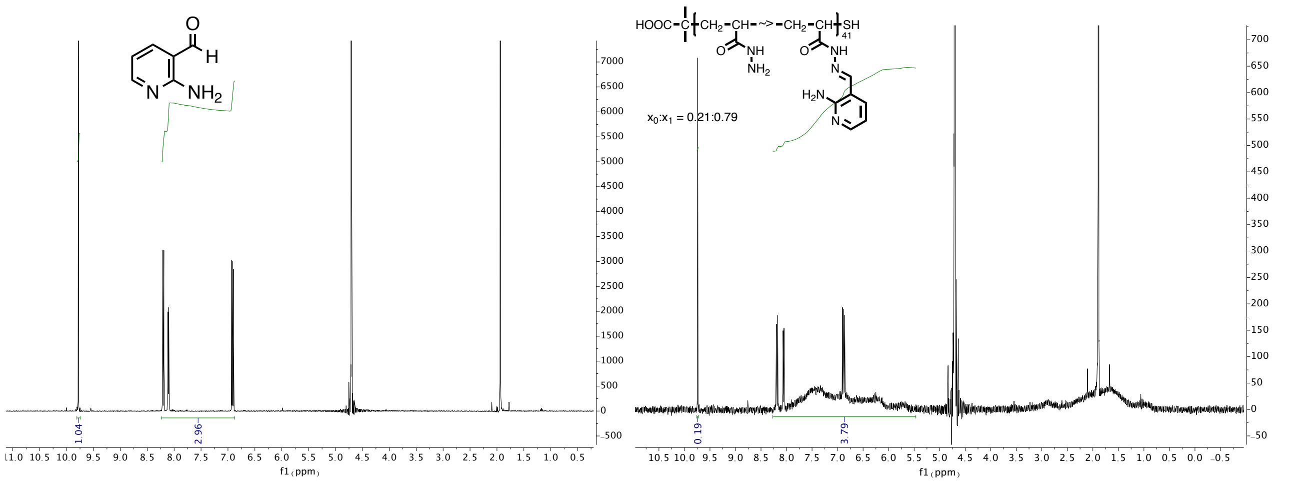

Figure S3: ¹H-NMRs of **2AFPA** (left) before the coupling reaction and **P1-*mod*-2AFPA** (right).

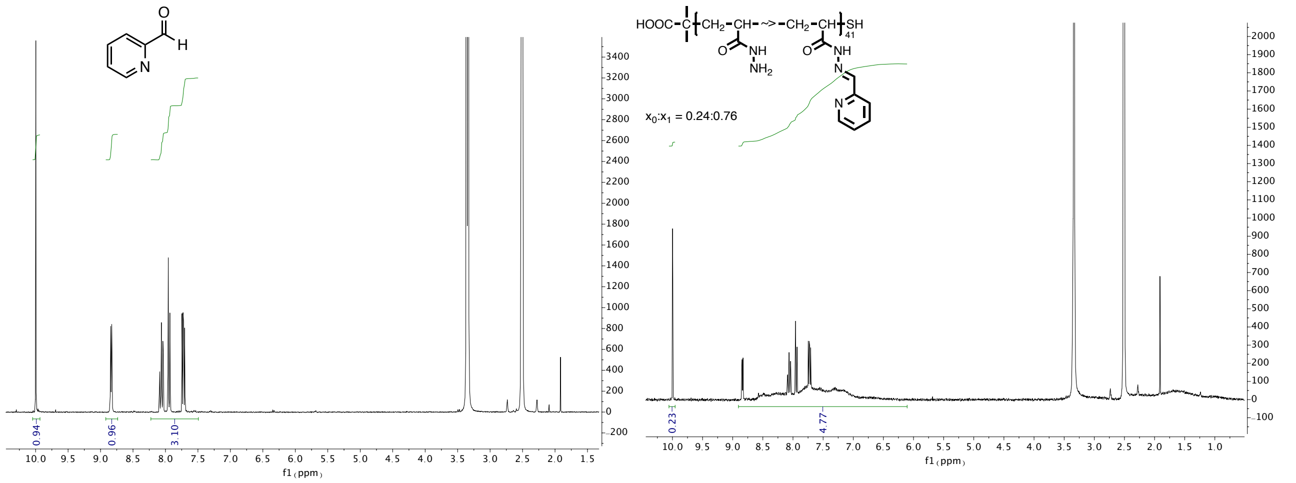

Figure S4: ¹H-NMRs of **2PyA** (left) before the coupling reaction and **P1-*mod*-2PyA** (right).

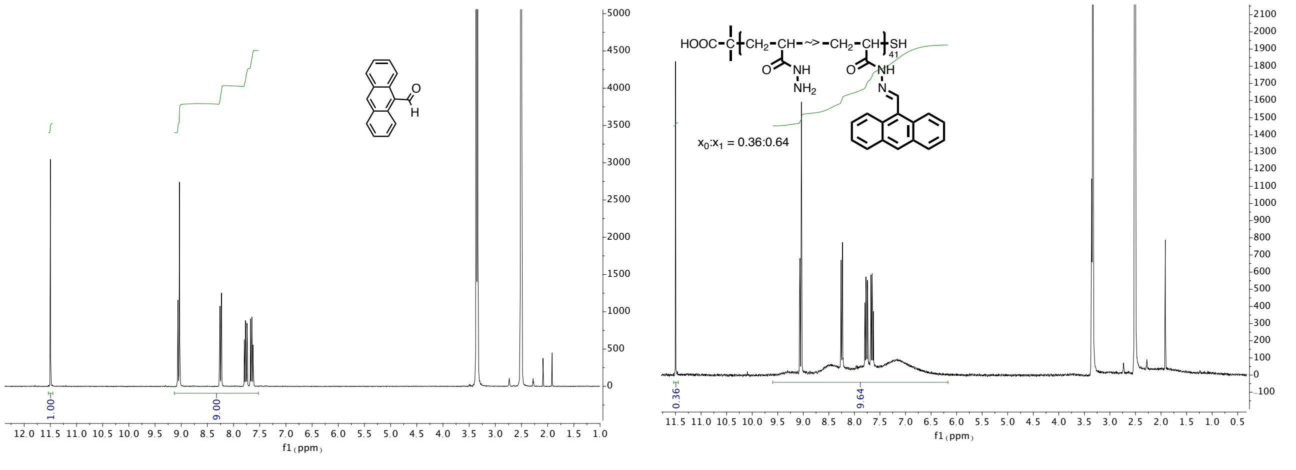

Figure S5: ¹H-NMRs of **9AntA** (left) before the coupling reaction and **P1-*mod*-9AntA** (right).

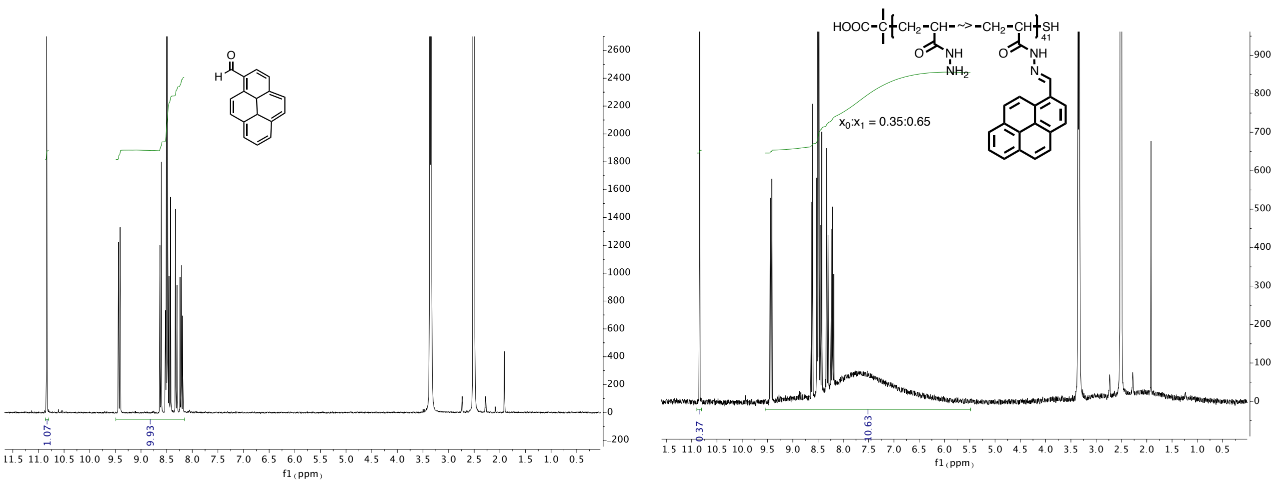

Figure S6: ^{1}H-NMRs of **1PyrA** (left) before the coupling reaction and **P1-*mod*-1PyrA** (right).

5.4. Calculation of water:Octanol partition coefficients at pH 7 (cLogD)

Representative monomer units were drawn in MarvinSketch® and cLogD calculated using the *LogD Predictor* plugin. clogD was then averaged per monomer unit as shown below. Because **P1-*mod*-OctA** was insoluble in the NMR mixture, we could not calculate degree of functionalization. Loading for this polymer was taken as the average of the loading for the other aldehydes, in order to include this polymer also in the graphs above.

Table S1 – Calculated water:Octanol partition coefficients at pH 7 (cLogD).

Polymer	clogD hydrazone	clogD Hydrazide	Fraction functionalised	clogD Polymer
P1		-0.08		-0.08
P1-*mod*-4ImA	0.89	-0.08	0.56	0.50
P1-*mod*-2AFPA	0.84	-0.08	0.79	0.66
P1-*mod*-2PyA	1.81	-0.08	0.76	1.38
P1-*mod*-3InA	2.45	-0.08	0.58	1.42
P1-*mod*-IvA	1.72	-0.08	0.9	1.55
P1-*mod*-BnA	2.35	-0.08	0.76	1.79
P1-*mod*-2NphA	3.34	-0.08	0.78	2.61
P1-*mod*-9AntA	4.33	-0.08	0.64	2.77
P1-*mod*-1PyrA	4.57	-0.08	0.65	2.97
P1-*mod*-OctA*	3.21	-0.08	0.71	2.29

*Fraction functionalised taken as the average of the values above.

6. Biofilm formation

6.1. Biofilm quantification using crystal violet

MC4100 was grown overnight at 30 °C in 10 mL LB. The morning after, the cells were centrifuged down, washed twice with water (2 x 10 mL) and resuspended to a final total volume of 1 mL in 15 mL falcon tubes as in section 3 above. Briefly, the pellet was resuspended in 0.1 M KH_2PO_4/K_2HPO_4 so that an OD_{600} of 0.2 was reached. Polymers were then added to the culture at 0.1 mg/mL, the culture supplemented with an equal volume of standard M63 minimal media and incubated at 30 °C, 150 rpm for 24 h, 48 h, 3 days or 5 days respectively. After incubation, the polymer-cell clusters (sedimented at the bottom of the falcon tube) were washed with water by taking out 900 µL of the suspension and gently adding back 900 µL of water. This procedure was repeated again with extra care to not disrupt the sedimented clusters. Using a table lamp to enhance visualisation is recommended.

After the second wash, 900 µL of supernatant was removed and 1 mL of a 1% crystal violet solution was added. A pipette tip was used to gently mix the crystal violet into the polymer-cells sediment and this suspension was left at r.t. for 1 h. The stained suspension was gently washed three times with water by taking out 800 µL of solution and adding back 800 µL of water, again ensuring not to disrupt the sediment. 20 min were left in between washes to reduce sediment disruption. After the final addition of water, the suspension was centrifuged down (30 s, 3900 rpm) and the supernatant removed. The pellet was then resuspended in 2 mL of 33% acetic acid and further diluted by adding 3 mL water and the absorbance (550 nm) of the solutions then measured. Aldehyde controls were performed in the exact same way using the amount of aldehyde used to functionalize **P1**.

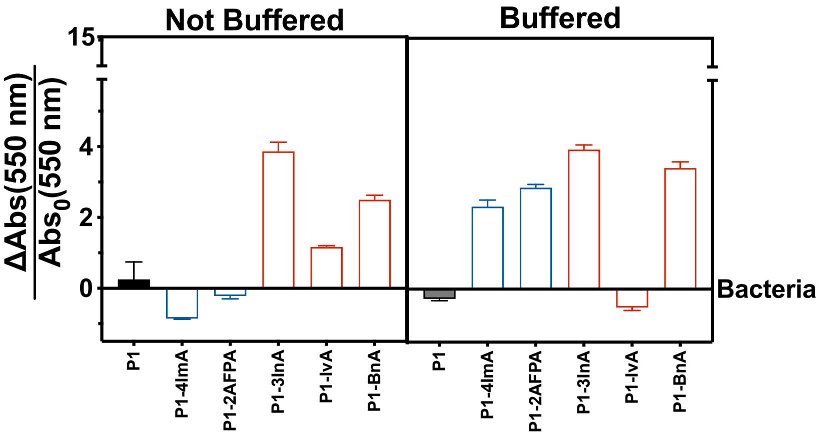

Figure S7 – Biofilm formation as measured by crystal violet staining: Fractional change in absorbance at 550 nm for *E. coli* PHL644 cultures following incubation over 3 days in the presence of 0.05 mg/mL of **P1** and functional polymers **P1-*mod*-aldehyde**. Data has been normalised and represents the fractional change in absorbance at 550 nm when compared to *E. coli* PHL644 cultures incubated in the absence of polymers (solid line). Not buffered indicates incubation in 100 mM aqueous NaCl. Buffered indicates incubation in 100 mM phosphate buffer at pH 7. Means ± range from at least three biological replicates are shown.

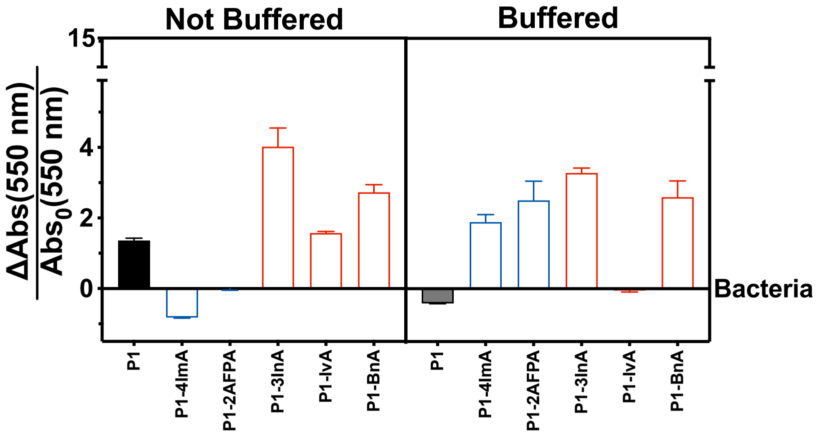

Figure S8 – Biofilm formation as measured by crystal violet staining: Fractional change in absorbance at 550 nm for *E. coli* PHL644 cultures following incubation over 5 days in the presence of 0.05 mg/mL of **P1** and functional polymers **P1-*mod*-aldehyde**. Data has been normalised and represents the fractional change in absorbance at 550 nm when compared to *E. coli* PHL644 cultures incubated in the absence of polymers (solid line). Not buffered indicates incubation in 100 mM aqueous NaCl. Buffered indicates incubation in 100 mM phosphate buffer at pH 7. Means ± range from at least three biological replicates are shown.

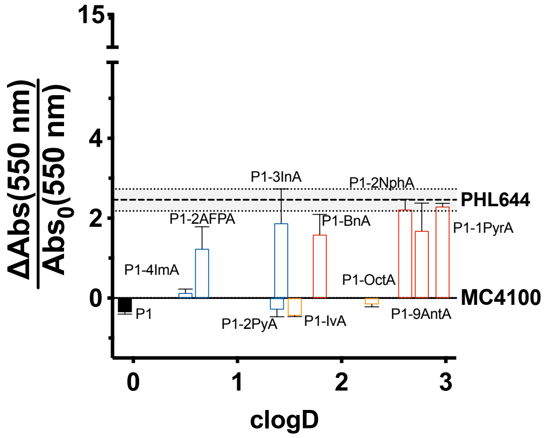

Figure S9 – Biofilm formation as measured by crystal violet staining: Fractional change in absorbance at 550 nm for *E. coli* MC4100 cultures following incubation in 100 mM phosphate buffer at pH 7 over 48 h in the presence of 0.05 mg/mL of **P1** (black solid bar) and functional polymers **P1-*mod*-aldehyde** (hollow coloured bars). Data has been normalised and represents the fractional change in absorbance at 550 nm when compared to *E. coli* MC4100 cultures incubated in the absence of polymers (solid line). Fractional change in absorbance at 550 nm for *E. coli* PHL644 cultures incubated in the absence of polymers when compared to *E. coli* MC4100 cultures incubated in the absence of polymers is also shown for comparison (dashed line). Means ± range from at least three biological replicates are shown.

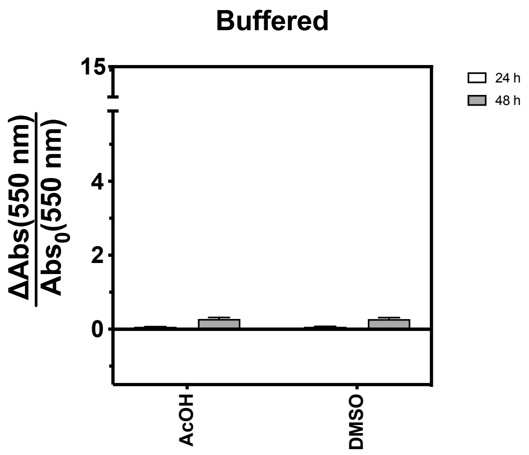

Figure S10 – Biofilm formation as measured by crystal violet staining: Fractional change in absorbance at 550 nm for *E. coli* MC4100 cultures following incubation in 100 mM phosphate buffer at pH 7 with an equivalent amount of 100 mM acetic acid (AcOH) or 95% DMSO-d6/5% 100 mM acetic acid (DMSO). Data has been normalised and represents the fractional change in absorbance at 550 nm when compared to *E. coli* MC4100 cultures incubated in the absence of polymers (solid line). Buffered indicates incubation in 100 mM phosphate buffer at pH 7. Means ± range from at least three biological replicates are shown.

6.2. Curli expression using GFP reporter

Strains used in this experiment had been transformed with pJLC-T.[4] Polymer-cell suspensions were prepared as described in section 3 above. Briefly, the pellet was resuspended in 0.1 M KH_2PO_4/K_2HPO_4 so that an OD_{600} of 0.2 was reached. Polymers were then added to the culture at 0.1 mg/mL, the culture supplemented with an equal volume of standard M63 minimal media – for this assay, growth media was supplemented with 10 μg/mL tetracycline – and placed in an flat bottom clear 96 well plate to a final volume of 300 μL. The plate was put into a BMG Labtech CLARIOstar Plus microplate reader where it was incubated at 30 °C with shaking at 100 rpm in orbital mode. During this time, fluorescence emission was measured every hour for 48 h using the preset GFP excitation/emission values (λ_{exc} = 488 nm, λ_{em} = 510 nm) . GFP expression was normalized to minimize error between independent biological replicates. First, GFP fluorescence at *t0* for *E. coli* cultures in the absence of polymers was subtracted for each respective sample. Then, samples were

normalised so that the maximum value of GFP fluorescence for *E. coli* cultures in the absence of polymers was 1 (**Figure S11-Figure S23**). The total amount of GFP fluorescence (**Figure 3B**) was determined by calculating the area under the graph. Onset of fluorescence (**Figure S24**) and rate of curli expression (**Figure 3C**) were calculated by fitting GFP fluorescence before the maximum value to a segmental line regression. These fittings were done using GraphPad Prism version 8.0.0, GraphPad Software, San Diego, California USA, www.graphpad.com.

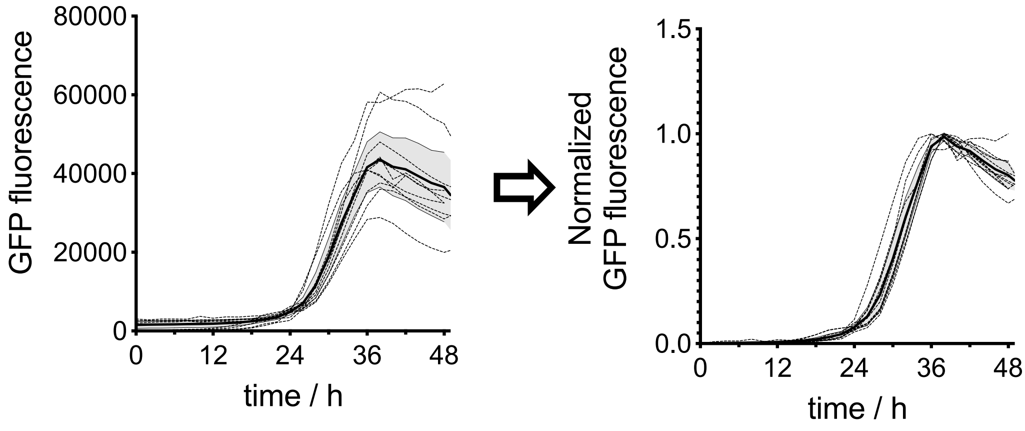

Figure S11 – Curli expression measured using a GFP reporter strain - Normalization: GFP fluorescence (left) and Normalized GFP fluorescence (right) for *E. coli* MC4100 pJLC-T cultures following incubation in 100 mM phosphate buffer at pH 7. Means (solid line) ± 95% confidence intervals (shadowed area) and individual values (dashed lines) are shown. N=10.

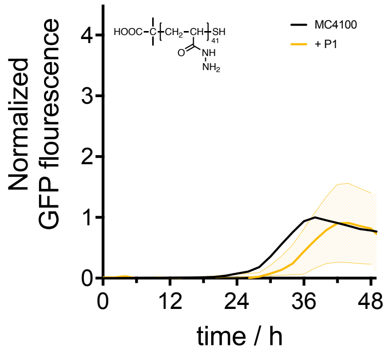

Figure S12 – Curli expression measured using a GFP reporter strain: Normalized GFP fluorescence for *E. coli* MC4100 pJLC-T cultures following incubation in 100 mM phosphate buffer at pH 7 in the absence (black, n=10) and presence of 0.05 mg/mL of **P1** (yellow, n=8). Means ± 95% confidence intervals are shown.

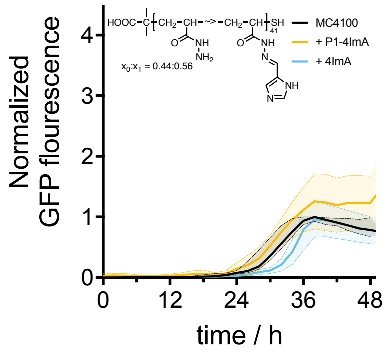

Figure S13 – Curli expression measured using a GFP reporter strain: Normalized GFP fluorescence for *E. coli* MC4100 pJLC-T cultures following incubation in 100 mM phosphate buffer at pH 7 in the absence (black, n=10) and presence of 0.05 mg/mL of **P1-*mod*-4ImA** (yellow, n=8) or **4ImA** (blue, n=3). Means ± 95% confidence intervals shown.

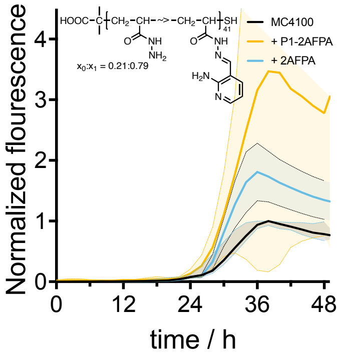

Figure S14 – Curli expression measured using a GFP reporter strain: Normalized GFP fluorescence for *E. coli* MC4100 pJLC-T cultures following incubation in 100 mM phosphate buffer at pH 7 in the absence (black, n=10) and presence of 0.05 mg/mL of **P1-*mod*-2AFPA** (yellow, n=8) or **2AFPA** (blue, n=3). Means ± 95% confidence intervals are shown.

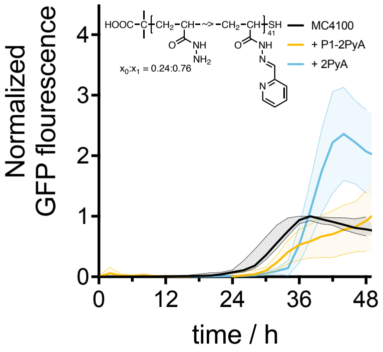

Figure S15 – Curli expression measured using a GFP reporter strain: Normalized GFP fluorescence for *E. coli* MC4100 pJLC-T cultures following incubation in 100 mM phosphate buffer at pH 7 in the absence (black, n=10) and presence of 0.05 mg/mL of **P1-*mod*-2PyA** (yellow, n=6) or **2PyA** (blue, n=3). Means ± 95% confidence intervals are shown.

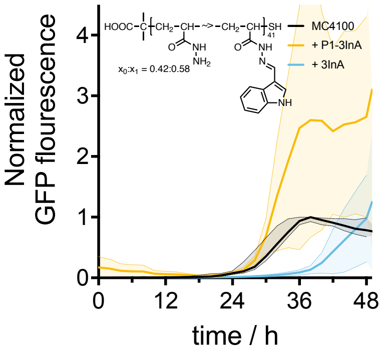

Figure S16 – Curli expression measured using a GFP reporter strain: Normalized GFP fluorescence for *E. coli* MC4100 pJLC-T cultures following incubation in 100 mM phosphate buffer at pH 7 in the absence (black, n=10) and presence of 0.05 mg/mL of **P1-*mod*-3InA** (yellow, n=10) or **3InA** (blue, n=5). Means ± 95% confidence intervals are shown.

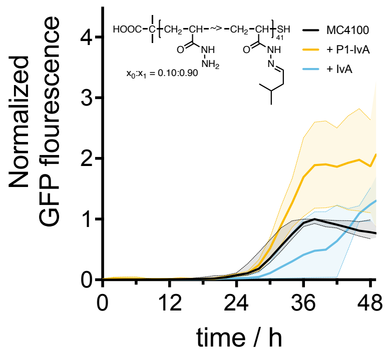

Figure S17 – Curli expression measured using a GFP reporter strain: Normalized GFP fluorescence for *E. coli* MC4100 pJLC-T cultures following incubation in 100 mM phosphate buffer at pH 7 in the absence (black, n=10) and presence of 0.05 mg/mL of **P1-*mod*-IvA** (yellow, n=10) or **IvA** (blue, n=5). Means ± 95% confidence intervals are shown.

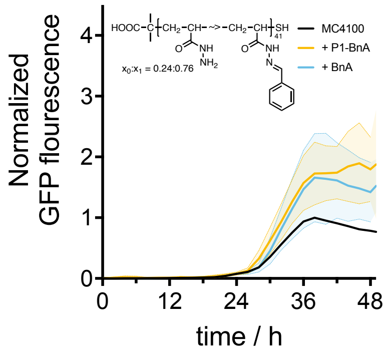

Figure S18 – Curli expression measured using a GFP reporter strain: Normalized GFP fluorescence for *E. coli* MC4100 pJLC-T cultures following incubation in 100 mM phosphate buffer at pH 7 in the absence (black, n=10) and presence of 0.05 mg/mL of **P1-*mod*-BnA** (yellow, n=10) or **BnA** (blue, n=5). Means ± 95% confidence intervals are shown.

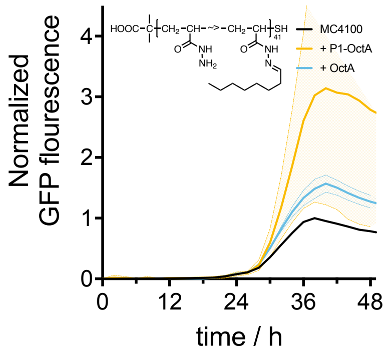

Figure S19 – Curli expression measured using a GFP reporter strain: Normalized GFP fluorescence for *E. coli* MC4100 pJLC-T cultures following incubation in 100 mM phosphate buffer at pH 7 in the absence (black, n=10) and presence of 0.05 mg/mL of **P1-*mod*-OctA** (yellow, n=6) or **OctA** (blue, n=3). Means ± 95% confidence intervals are shown.

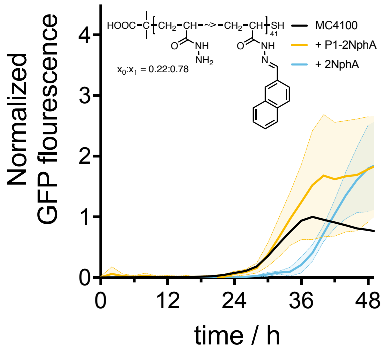

Figure S20 – Curli expression measured using a GFP reporter strain: Normalized GFP fluorescence for *E. coli* MC4100 pJLC-T cultures following incubation in 100 mM phosphate buffer at pH 7 in the absence (black, n=10) and presence of 0.05 mg/mL of **P1-*mod*-2NphA** (yellow, n=6) or **2NphA** (blue, n=3). Means ± 95% confidence intervals are shown.

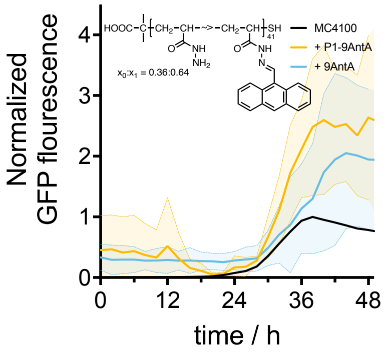

Figure S21 – Curli expression measured using a GFP reporter strain: Normalized GFP fluorescence for *E. coli* MC4100 pJLC-T cultures following incubation in 100 mM phosphate buffer at pH 7 in the absence (black, n=10) and presence of 0.05 mg/mL of **P1-*mod*-9AntA** (yellow, n=6) or **9AntA** (blue, n=3). Means ± 95% confidence intervals from at least three biological replicates are shown.

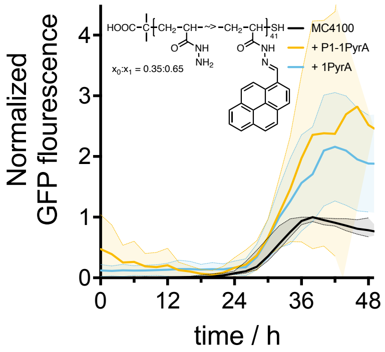

Figure S22 – Curli expression measured using a GFP reporter strain: Normalized GFP fluorescence for *E. coli* MC4100 pJLC-T cultures following incubation in 100 mM phosphate buffer at pH 7 in the absence (black, n=10) and presence of 0.05 mg/mL of **P1-*mod*-1PyrA** (yellow, n=6) or **1PyrA** (blue, n=3). Means ± 95% confidence intervals are shown.

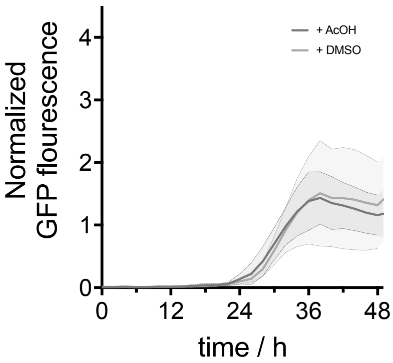

Figure S23 – Curli expression measured using a GFP reporter strain: Normalized GFP fluorescence for *E. coli* MC4100 pJLC-T cultures following incubation in 100 mM phosphate buffer at pH 7 with an equivalent amount of 100 mM acetic acid (AcOH) or 95% DMSO-d6/5% 100 mM acetic acid (DMSO). Means ± 95% confidence intervals are shown. N=8.

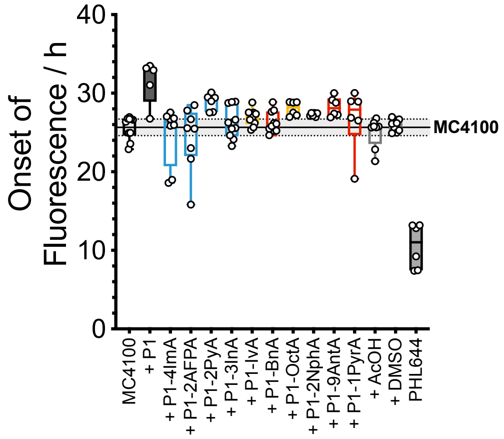

Figure S24 – Curli expression measured using a GFP reporter strain: Onset of fluorescence for *E. coli* MC4100 pJLC-T cultures following incubation in 100 mM phosphate buffer at pH 7 in the absence (black hollowed box) and presence of 0.05 mg/mL of **P1** (black solid box) and functional polymers **P1-*mod*-aldehyde** (coloured hollowed boxes). Onset of fluorescence for *E. coli* PHL644 pJLC-T cultures (grey solid box) shown for comparison. Median is shown as a line. Box extends from 25th to 75th percentile while whiskers go from minimum to maximum value.

7. Aggregation of bacteria

7.1. Spectrophotometric cell clustering

MC4100 was grown overnight in 10 mL LB. The morning after, the cells were centrifuged down and washed twice with water (2 x 10 mL). The pellet was resuspended in 0.1 M KH_2PO_4/K_2HPO_4 to give an OD_{600} of 1. The culture was split into 1 mL aliquots in 1 mL plastic cuvettes. Polymers were then added respectively to a final concentration of 0.5 mg/mL and OD_{600} was measured immediately, then every 30 min for 4 h and finally once at 24 h whilst incubating at 30 °C, 150 rpm between measurements. An identical experiment was performed using the respective aldehydes (0.5 mg/mL) rather than adding polymer to serve as a control. Further control experiments without bacteria were also performed, whereby the respective polymers were added to 0.1 M KH_2PO_4/K_2HPO_4 to a final concentration of 0.05 mg/mL and OD_{600} measured in the same way.

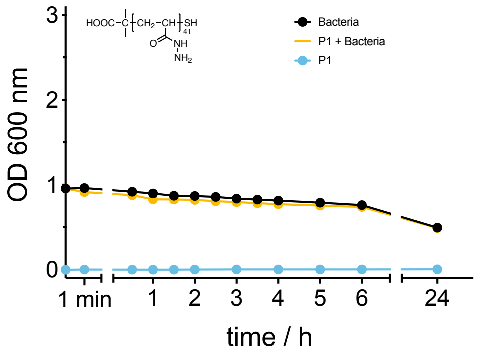

Figure S25 – Aggregation of bacteria: Optical density at 600 nm for *E. coli* MC4100 cultures following incubation for 24 h in the absence (black) and presence of 0.5 mg/mL of **P1** (yellow). Optical density of **P1** suspended in culture media (blue) shown for comparison. Means ± SD from at least two biological replicates are shown.

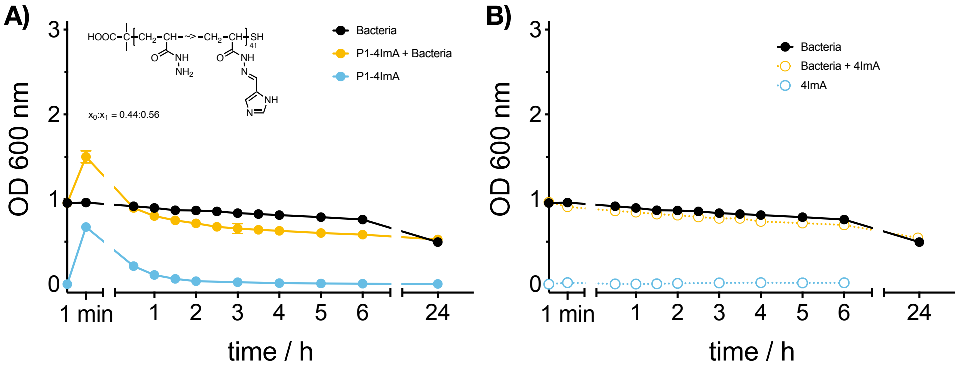

Figure S26 – Aggregation of bacteria: A) Optical density at 600 nm for *E. coli* MC4100 cultures following incubation for 24 h in the absence (black) and presence of 0.5 mg/mL of **P1-*mod*-4ImA** (yellow). Optical density of **P1-*mod*-4ImA** suspended in culture media (blue) shown for comparison. B) Optical density at 600 nm for *E. coli* MC4100 cultures following incubation for 24 h in the absence (black) and presence of 0.5 mg/mL of **4ImA** (yellow). Optical density of **4ImA** suspended in culture media (blue) shown for comparison. Means ± SD from at least two biological replicates are shown.

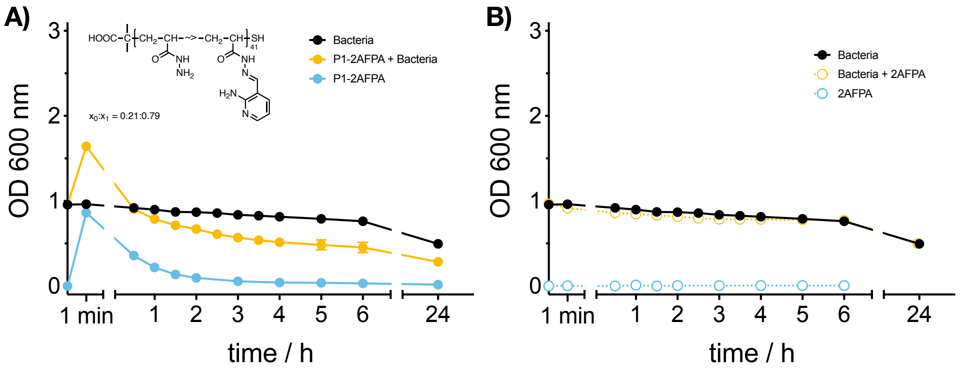

Figure S27 – Aggregation of bacteria: A) Optical density at 600 nm for *E. coli* MC4100 cultures following incubation for 24 h in the absence (black) and presence of 0.5 mg/mL of **P1-*mod*-2AFPA** (yellow). Optical density of **P1-*mod*-2AFPA** suspended in culture media (blue) shown for comparison. B) Optical density at 600 nm for *E. coli* MC4100 cultures following incubation for 24 h in the absence (black) and presence of 0.5 mg/mL of **2AFPA** (yellow). Optical density of **2AFPA** suspended in culture media (blue) shown for comparison. Means ± SD from at least two biological replicates are shown.

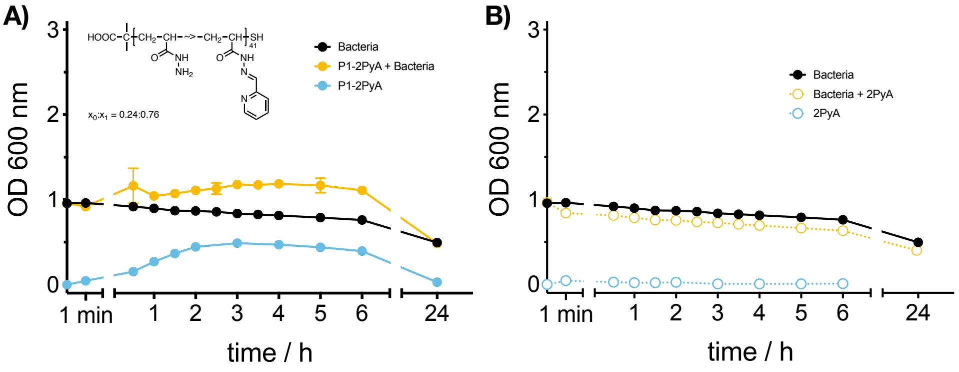

Figure S28 – Aggregation of bacteria: A) Optical density at 600 nm for *E. coli* MC4100 cultures following incubation for 24 h in the absence (black) and presence of 0.5 mg/mL of **P1-*mod*-2PyA** (yellow). Optical density of **P1-*mod*-2PyA** suspended in culture media (blue) shown for comparison. B) Optical density at 600 nm for *E. coli* MC4100 cultures following incubation for 24 h in the absence (black) and presence of 0.5 mg/mL of **2PyA** (yellow). Optical density of **2PyA** suspended in culture media (blue) shown for comparison. Means ± SD from at least two biological replicates are shown.

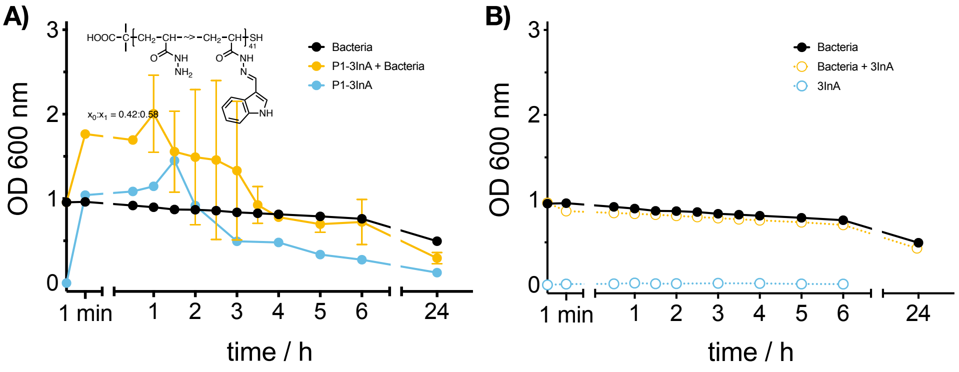

Figure S29 – Aggregation of bacteria: A) Optical density at 600 nm for *E. coli* MC4100 cultures following incubation for 24 h in the absence (black) and presence of 0.5 mg/mL of **P1-*mod*-3InA** (yellow). Optical density of **P1-*mod*-3InA** suspended in culture media (blue) shown for comparison. B) Optical density at 600 nm for *E. coli* MC4100 cultures following incubation for 24 h in the absence (black) and presence of 0.5 mg/mL of **3InA** (yellow). Optical density of **3InA** suspended in culture media (blue) shown for comparison. Means ± SD from at least two biological replicates are shown.

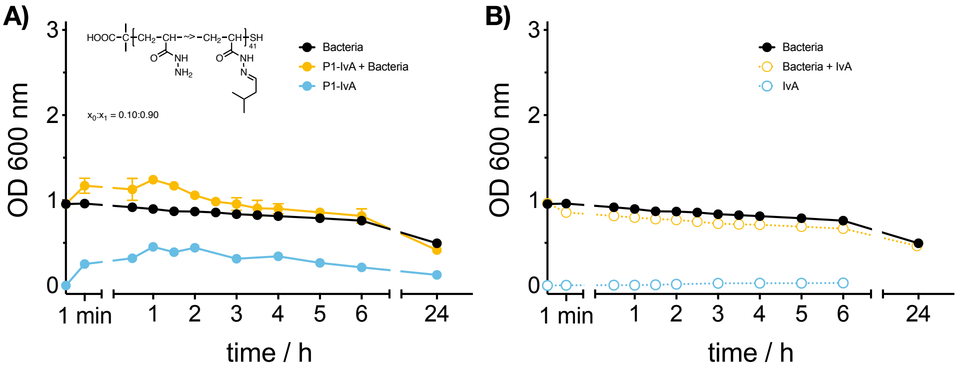

Figure S30 – Aggregation of bacteria: A) Optical density at 600 nm for *E. coli* MC4100 cultures following incubation for 24 h in the absence (black) and presence of 0.5 mg/mL of **P1-*mod*-IvA** (yellow). Optical density of **P1-*mod*-IvA** suspended in culture media (blue) shown for comparison. B) Optical density at 600 nm for *E. coli* MC4100 cultures following incubation for 24 h in the absence (black) and presence of 0.5 mg/mL of **IvA** (yellow). Optical density of **IvA** suspended in culture media (blue) shown for comparison. Means ± SD from at least two biological replicates are shown.

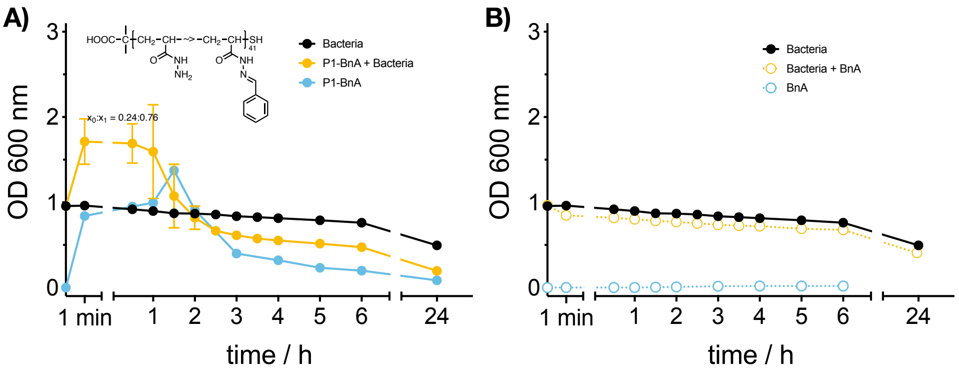

Figure S31 – Aggregation of bacteria: A) Optical density at 600 nm for *E. coli* MC4100 cultures following incubation for 24 h in the absence (black) and presence of 0.5 mg/mL of **P1-*mod*-BnA** (yellow). Optical density of **P1-*mod*-BnA** suspended in culture media (blue) shown for comparison. B) Optical density at 600 nm for *E. coli* MC4100 cultures following incubation for 24 h in the absence (black) and presence of 0.5 mg/mL of **BnA** (yellow). Optical density of **BnA** suspended in culture media (blue) shown for comparison. Means ± SD from at least two biological replicates are shown.

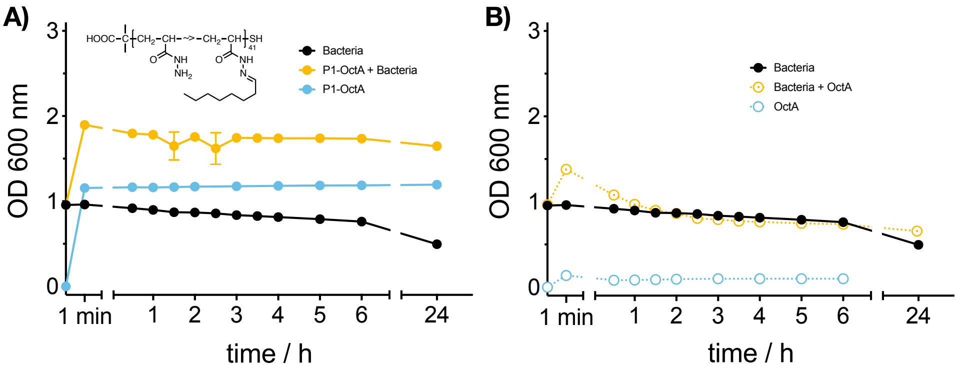

Figure S32 – Aggregation of bacteria: A) Optical density at 600 nm for *E. coli* MC4100 cultures following incubation for 24 h in the absence (black) and presence of 0.5 mg/mL of **P1-*mod*-OctA** (yellow). Optical density of **P1-*mod*-OctA** suspended in culture media (blue) shown for comparison. B) Optical density at 600 nm for *E. coli* MC4100 cultures following incubation for 24 h in the absence (black) and presence of 0.5 mg/mL of **OctA** (yellow). Optical density of **OctA** suspended in culture media (blue) shown for comparison. Means ± SD from at least two biological replicates are shown.

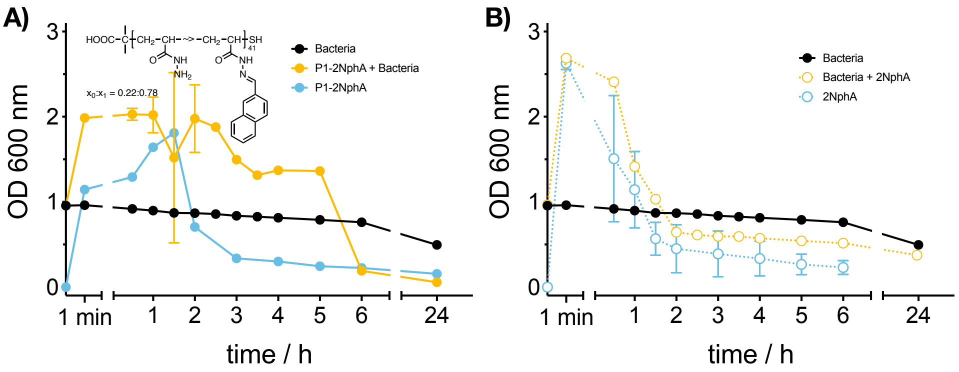

Figure S33 – Aggregation of bacteria: A) Optical density at 600 nm for *E. coli* MC4100 cultures following incubation for 24 h in the absence (black) and presence of 0.5 mg/mL of **P1-*mod*-2NphA** (yellow). Optical density of **P1-*mod*-2NphA** suspended in culture media (blue) shown for comparison. B) Optical density at 600 nm for *E. coli* MC4100 cultures following incubation for 24 h in the absence (black) and presence of 0.5 mg/mL of **2NphA** (yellow). Optical density of **2NphA** suspended in culture media (blue) shown for comparison. Means ± SD from at least two biological replicates are shown.

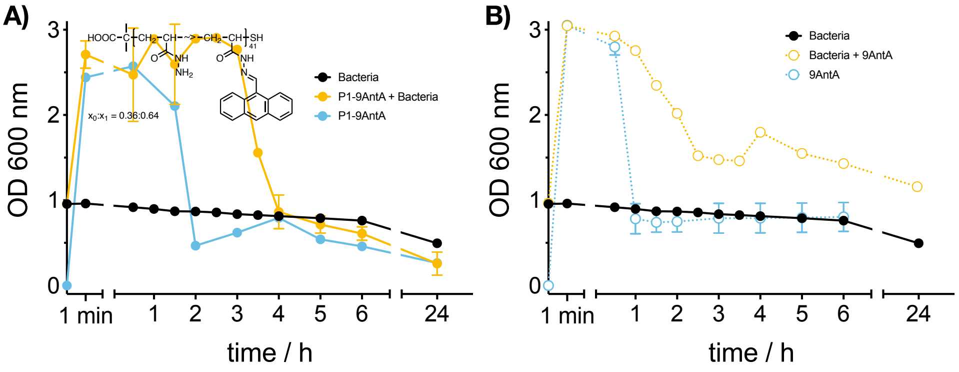

Figure S34 – Aggregation of bacteria: A) Optical density at 600 nm for *E. coli* MC4100 cultures following incubation for 24 h in the absence (black) and presence of 0.5 mg/mL of **P1-*mod*-9AntA** (yellow). Optical density of **P1-*mod*-9AntA** suspended in culture media (blue) shown for comparison. B) Optical density at 600 nm for *E. coli* MC4100 cultures following incubation for 24 h in the absence (black) and presence of 0.5 mg/mL of **9AntA** (yellow). Optical density of **9AntA** suspended in culture media (blue) shown for comparison. Means ± SD from at least two biological replicates are shown.

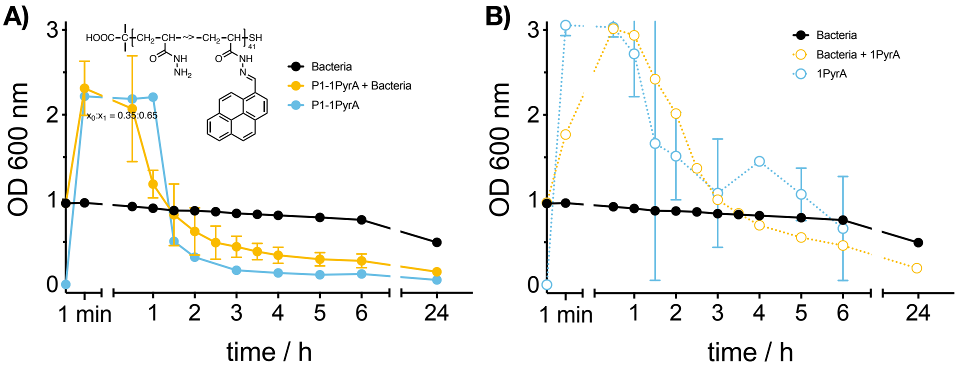

Figure S35 – Aggregation of bacteria: A) Optical density at 600 nm for *E. coli* MC4100 cultures following incubation for 24 h in the absence (black) and presence of 0.5 mg/mL of **P1-*mod*-1PyrA** (yellow). Optical density of **P1-*mod*-1PyrA** suspended in culture media (blue) shown for comparison. B) Optical density at 600 nm for *E. coli* MC4100 cultures following incubation for 24 h in the absence (black) and presence of 0.5 mg/mL of **1PyrA** (yellow). Optical density of **1PyrA** suspended in culture media (blue) shown for comparison. Means ± SD from at least two biological replicates are shown.

7.2. Sizing of polymer-bacteria aggregates

Polymer-cell suspensions were prepared as detailed in section 3 above. Briefly, the pellet was resuspended in 0.1 M KH_2PO_4/K_2HPO_4 so that an OD_{600} of 0.2 was reached. Polymers were then added to the culture at 0.1 mg/mL, the culture supplemented with an equal volume of standard M63 minimal media. For this assay, cells were resuspended to a final total volume of 2 mL in 15 mL falcon tubes and incubated at 30 °C, 150 rpm for 48 h respectively. Polymer-cell cluster sizes were analysed using a Malvern Mastersizer 2000. Plastic pipettes were used to carefully transfer the whole 2 mL suspensions respectively into the Mastersizer dispersion chamber filled with 100 mL water mixing at 500 rpm, the obscuration was set to 1% and the cluster sizes were measured whilst mixing. To determine the maximum peak sizes, particle size distributions were deconvoluted using the peakfit.m command. Percentage of free bacteria was calculated from cumulative plots calculating the percentage volume between 0.5 and 2 µm.

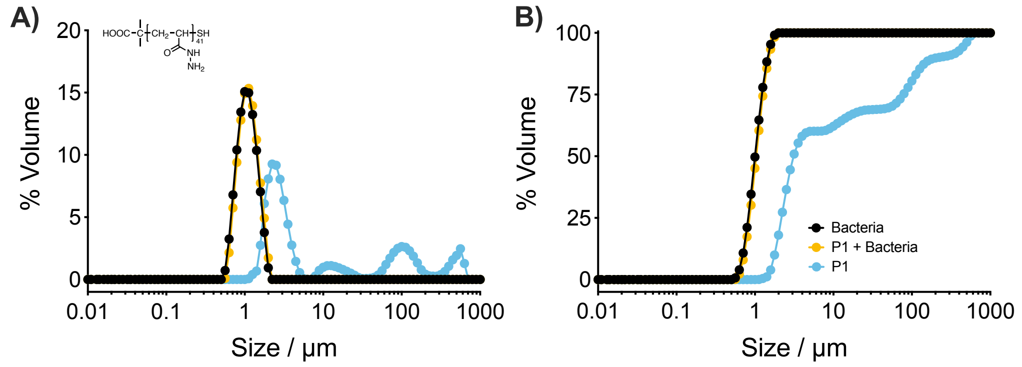

Figure S36 – Aggregation of bacteria: A) Size distribution and B) cumulative size distributions of suspensions of MC4100 cultures (black) and *E. coli* MC4100 cultures in the presence of 0.05 mg/mL of **P1** (yellow), following incubation over 48 h. Size distribution of suspensions of **P1** in culture media (blue) shown for comparison. Means ± SD from at least two biological replicates are shown.

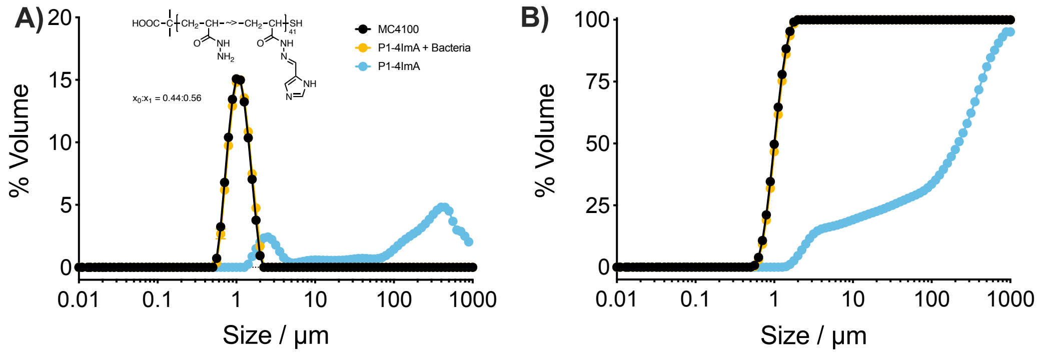

Figure S37 – Aggregation of bacteria: A) Size distribution and B) cumulative size distributions of suspensions of MC4100 cultures (black) and *E. coli* MC4100 cultures in the presence of 0.05 mg/mL of **P1-*mod*-4ImA** (yellow), following incubation over 48 h. Size distribution of suspensions of 0.05 mg/mL of **P1-*mod*-4ImA** in culture media (blue) shown for comparison. Means ± SD from at least two biological replicates are shown.

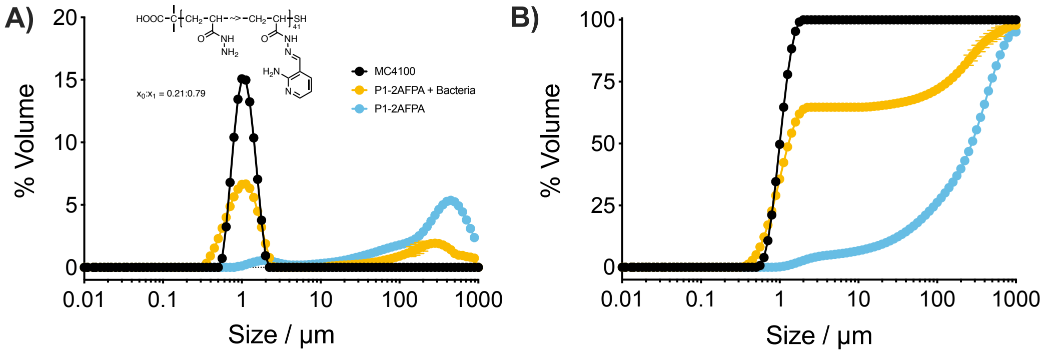

Figure S38 – Aggregation of bacteria: A) Size distribution and B) cumulative size distributions of suspensions of MC4100 cultures (black) and *E. coli* MC4100 cultures in the presence of 0.05 mg/mL of **P1-*mod*-2AFPA** (yellow), following incubation over 48 h. Size distribution of suspensions of 0.05 mg/mL of **P1-*mod*-2AFPA** in culture media (blue) shown for comparison. Means ± SD from at least two biological replicates are shown.

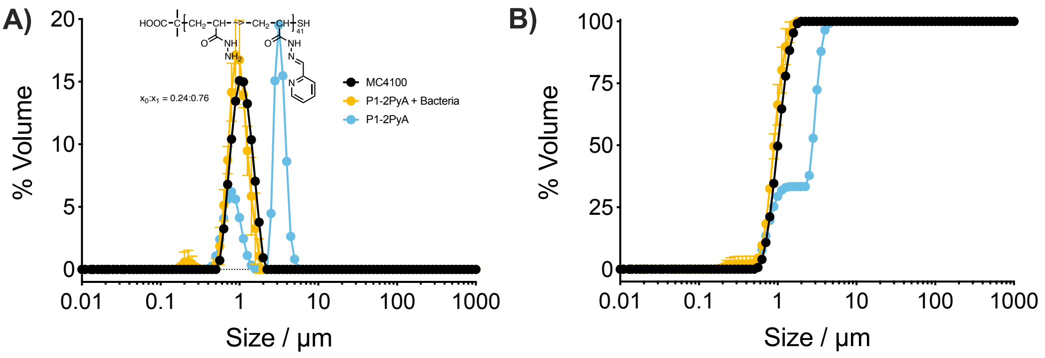

Figure S39 – Aggregation of bacteria: A) Size distribution and B) cumulative size distributions of suspensions of MC4100 cultures (black) and *E. coli* MC4100 cultures in the presence of 0.05 mg/mL of **P1-*mod*-2PyA** (yellow), following incubation over 48 h. Size distribution of suspensions of 0.05 mg/mL of **P1-*mod*-2PyA** in culture media (blue) shown for comparison. Means ± SD from at least two biological replicates are shown.

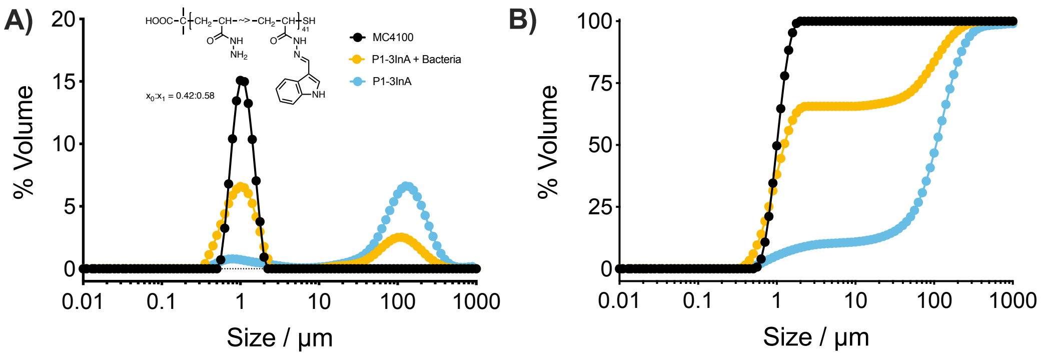

Figure S40 – Aggregation of bacteria: A) Size distribution and B) cumulative size distributions of suspensions of MC4100 cultures (black) and *E. coli* MC4100 cultures in the presence of 0.05 mg/mL of **P1-*mod*-3InA** (yellow), following incubation over 48 h. Size distribution of suspensions of 0.05 mg/mL of **P1-*mod*-3InA** in culture media (blue) shown for comparison. Means ± SD from at least two biological replicates are shown.

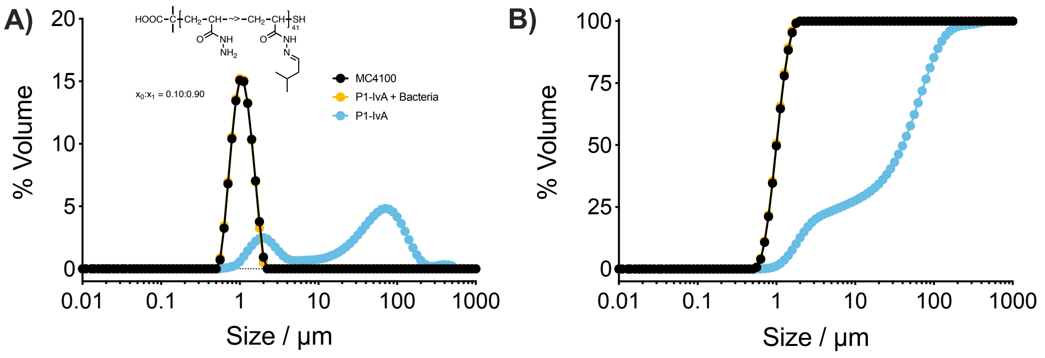

Figure S41 – Aggregation of bacteria: A) Size distribution and B) cumulative size distributions of suspensions of MC4100 cultures (black) and *E. coli* MC4100 cultures in the presence of 0.05 mg/mL of **P1-*mod*-IvA** (yellow), following incubation over 48 h. Size distribution of suspensions of 0.05 mg/mL of **P1-*mod*-IvA** in culture media (blue) shown for comparison. Means ± SD from at least two biological replicates are shown.

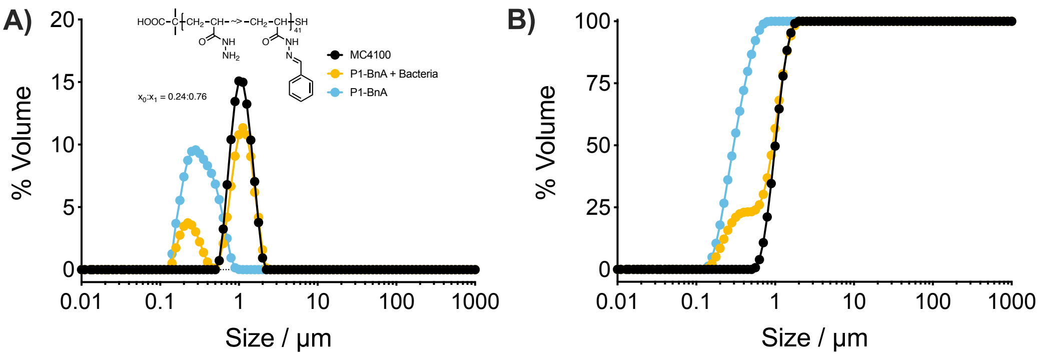

Figure S42 – Aggregation of bacteria: A) Size distribution and B) cumulative size distributions of suspensions of MC4100 cultures (black) and *E. coli* MC4100 cultures in the presence of 0.05 mg/mL of **P1-*mod*-BnA** (yellow), following incubation over 48 h. Size distribution of suspensions of 0.05 mg/mL of **P1-*mod*-BnA** in culture media (blue) shown for comparison. Means ± SD from at least two biological replicates are shown.

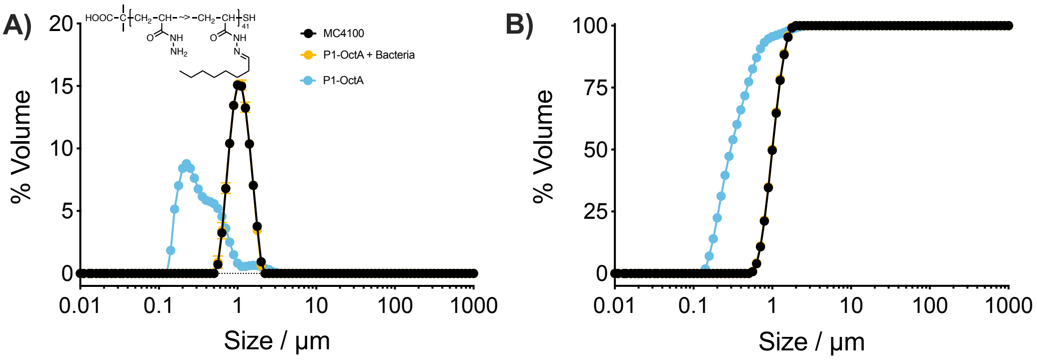

Figure S43 – Aggregation of bacteria: A) Size distribution and B) cumulative size distributions of suspensions of MC4100 cultures (black) and *E. coli* MC4100 cultures in the presence of 0.05 mg/mL of **P1-*mod*-OctA** (yellow), following incubation over 48 h. Size distribution of suspensions of 0.05 mg/mL of **P1-*mod*-OctA** in culture media (blue) shown for comparison. Means ± SD from at least two biological replicates are shown.

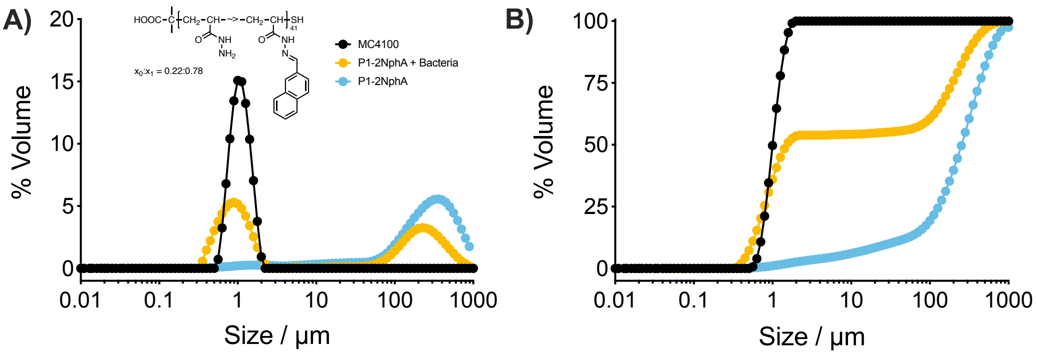

Figure S44 – Aggregation of bacteria: A) Size distribution and B) cumulative size distributions of suspensions of MC4100 cultures (black) and *E. coli* MC4100 cultures in the presence of 0.05 mg/mL of **P1-*mod*-2NphA** (yellow), following incubation over 48 h. Size distribution of suspensions of 0.05 mg/mL of **P1-*mod*-2NphA** in culture media (blue) shown for comparison. Means ± SD from at least two biological replicates are shown.

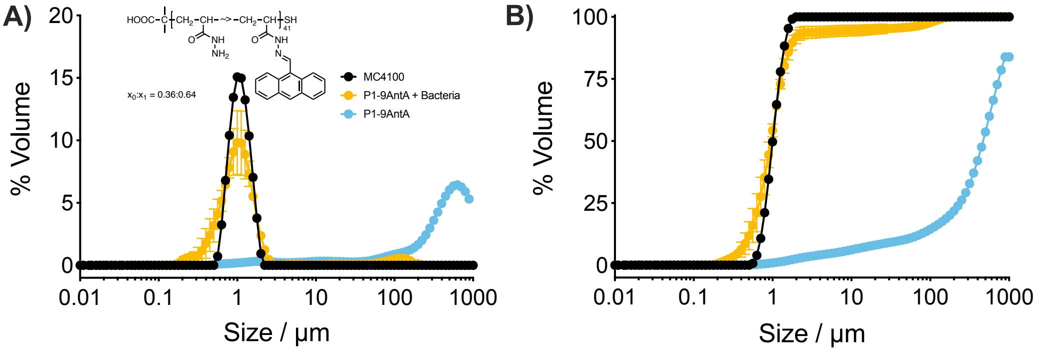

Figure S45 – Aggregation of bacteria: A) Size distribution and B) cumulative size distributions of suspensions of MC4100 cultures (black) and *E. coli* MC4100 cultures in the presence of 0.05 mg/mL of **P1-*mod*-9AntA** (yellow), following incubation over 48 h. Size distribution of suspensions of 0.05 mg/mL of **P1-*mod*-9AntA** in culture media (blue) shown for comparison. Means ± SD from at least two biological replicates are shown.

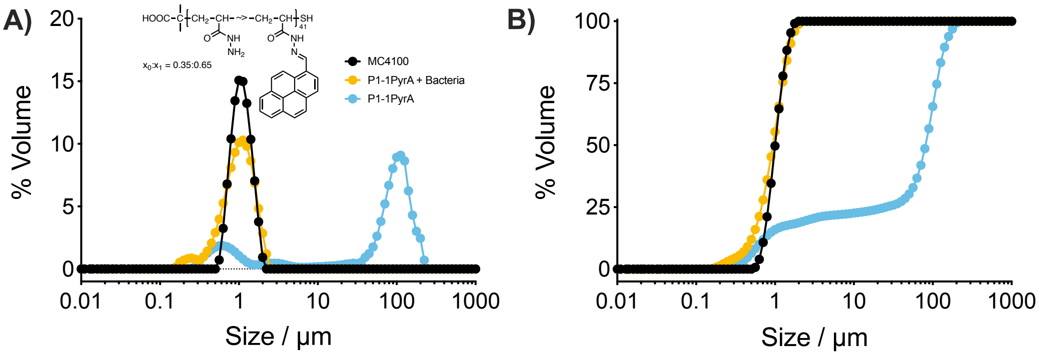

Figure S46 – Aggregation of bacteria: A) Size distribution and B) cumulative size distributions of suspensions of MC4100 cultures (black) and *E. coli* MC4100 cultures in the presence of 0.05 mg/mL of **P1-*mod*-1PyrA** (yellow), following incubation over 48 h. Size distribution of suspensions of 0.05 mg/mL of **P1-*mod*-1PyrA** in culture media (blue) shown for comparison. Means ± SD from at least two biological replicates are shown.

8. Biocatalysis: Synthesis of 5-fluorotryptophan

Polymer-cell suspensions (using *E. coli* transformed with pSTB7) were prepared as detailed in section 3 above. Briefly, the pellet was resuspended in 0.1 M KH_2PO_4/K_2HPO_4 so that an OD_{600} of 0.2 was reached. Polymers were then added to the culture at 0.1 mg/mL, the culture supplemented with an equal volume of standard M63 minimal media. For this assay, growth media was supplemented with 100 µg/mL ampicillin, cells were resuspended to a final volume of 1 mL in eppendorf tubes and incubated at 30 °C, 150 rpm for 48 h. After this incubation, the polymer-cell sediment was washed once by gently removing as much supernatant as possible (and measuring OD_{600}) without disrupting the sediment and adding back an equal volume of water. The supernatant was removed again and 1 mL of reaction buffer (0.1 M KH_2PO_4, 7 mM serine, 0.1 mM pyridoxal-5'-phosphate, 1 mM 5-fluoroindole adjusted to pH 7 with KOH and supplemented with 5% (v/v) DMSO) was added. The samples were placed back into an incubator at 30 °C, 150 rpm for 24 h after which the biotransformation was stopped by centrifugation (16000 g, 10 min). The sample supernatants (1 mL) were then filtered through a 0.45 µm PTFE filter and analysed by HPLC.

The relative concentrations of 5-fluoroindole were measured using HPLC by monitoring the decrease in sample peak integral corresponding to 5-fluoroindole from a 1 mM 5-fluoroindole control sample, and the relative increase in sample peak integral corresponding to 5-fluorotryptophan by comparing with the peak integral corresponding to 1 mM 5-fluorotryptophan sample in the reaction buffer (theoretical maximum yield from 1 mM 5-fluoroindole). Samples were analysed using a Shimadzu HPLC

with a C-18 column at 0.7 mL /min. A UV detector was used for the analysis. Gradient: Solvent A (Water 0.1% formic acid), Solvent B (Methanol 0.1% formic acid). 10% Solvent B, 30 s; 10-90% Solvent B over 12 min, hold 90% Solvent B for 2.5 min, 90-10% Solvent B over 1 min, hold 10% Solvent B for 5 min.

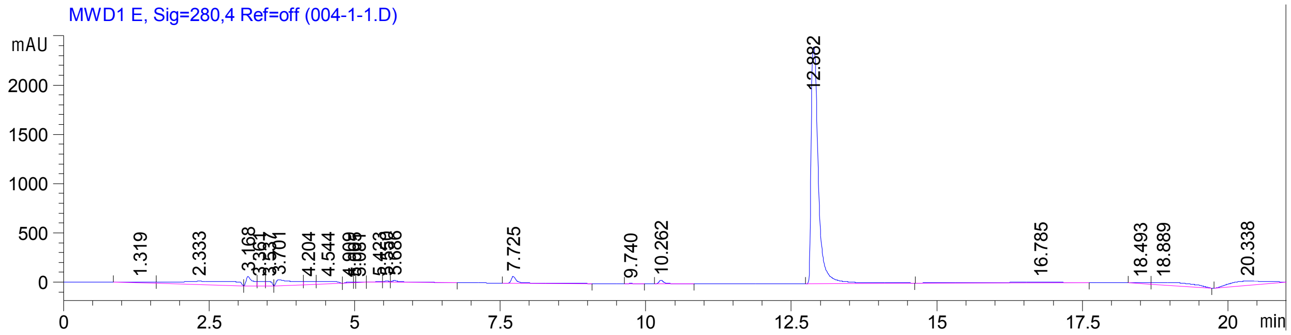

Figure S47 – Biocatalysis: HPLC chromatogram of filtered supernatant for *E. coli* MC4100 pSTB7 cultures following incubation in the presence of 0.05 mg/mL of **P1** for 24 h, followed by incubation with reaction buffer for another 24 h.

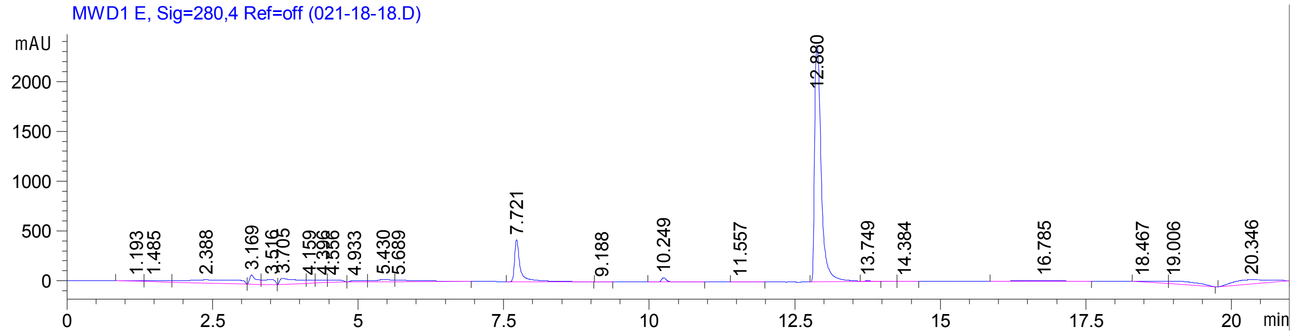

Figure S48 – Biocatalysis: HPLC chromatogram of filtered supernatant for *E. coli* MC4100 pSTB7 cultures following incubation in the presence of 0.05 mg/mL of **P1-*mod*-4ImA** for 24 h, followed by incubation with reaction buffer for another 24 h.

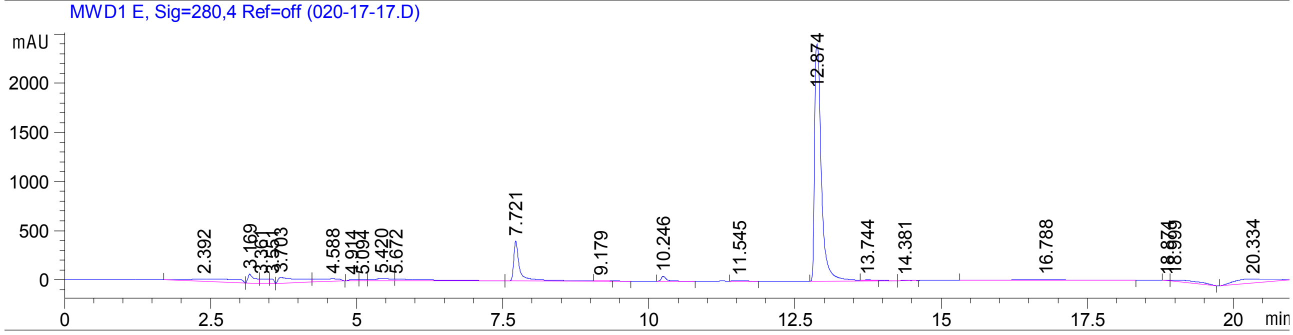

Figure S49 – Biocatalysis: HPLC chromatogram of filtered supernatant for *E. coli* MC4100 pSTB7 cultures following incubation in the presence of 0.05 mg/mL of **P1-*mod*-2AFPA** for 24 h, followed by incubation with reaction buffer for another 24 h.

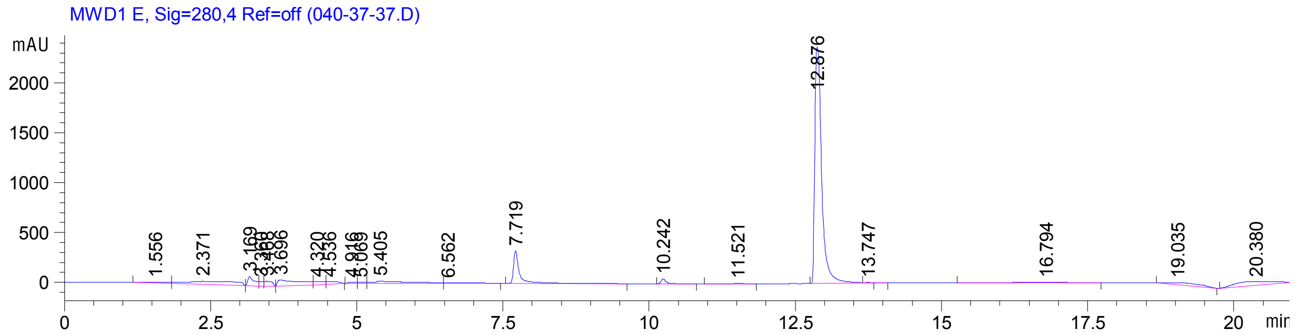

Figure S50 – Biocatalysis: HPLC chromatogram of filtered supernatant for *E. coli* MC4100 pSTB7 cultures following incubation in the presence of 0.05 mg/mL of **P1-*mod*-2PyA** for 24 h, followed by incubation with reaction buffer for another 24 h.

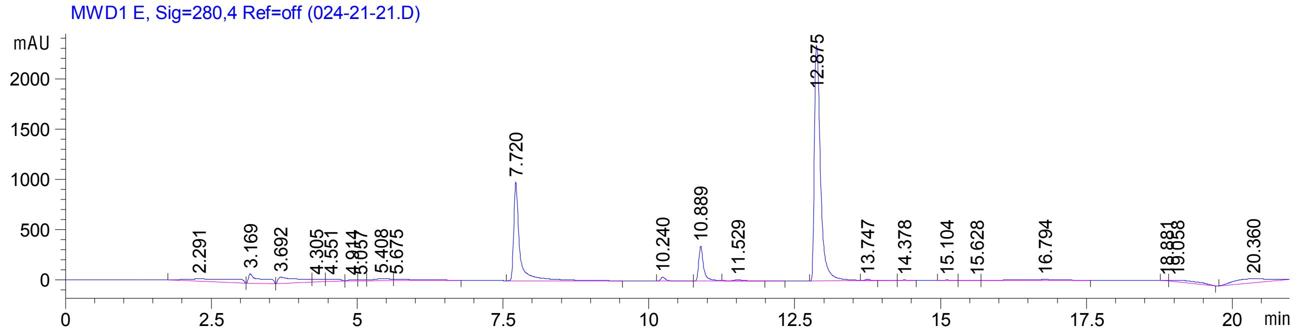

Figure S51 – Biocatalysis: HPLC chromatogram of filtered supernatant for *E. coli* MC4100 pSTB7 cultures following incubation in the presence of 0.05 mg/mL of **P1-*mod*-3InA** for 24 h, followed by incubation with reaction buffer for another 24 h.

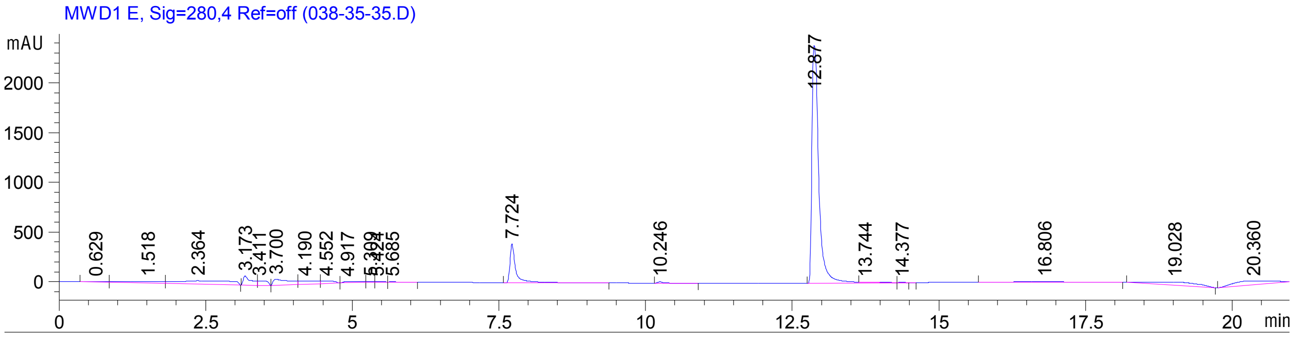

Figure S52 – Biocatalysis: HPLC chromatogram of filtered supernatant for *E. coli* MC4100 pSTB7 cultures following incubation in the presence of 0.05 mg/mL of **P1-*mod*-IvA** for 24 h, followed by incubation with reaction buffer for another 24 h.

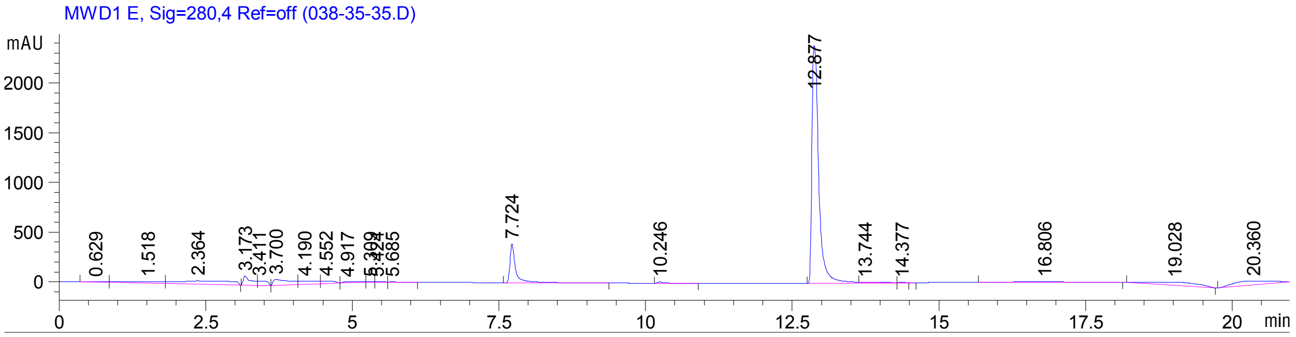

Figure S53 – Biocatalysis: HPLC chromatogram of filtered supernatant for *E. coli* MC4100 pSTB7 cultures following incubation in the presence of 0.05 mg/mL of **P1-*mod*-BnA** for 24 h, followed by incubation with reaction buffer for another 24 h.

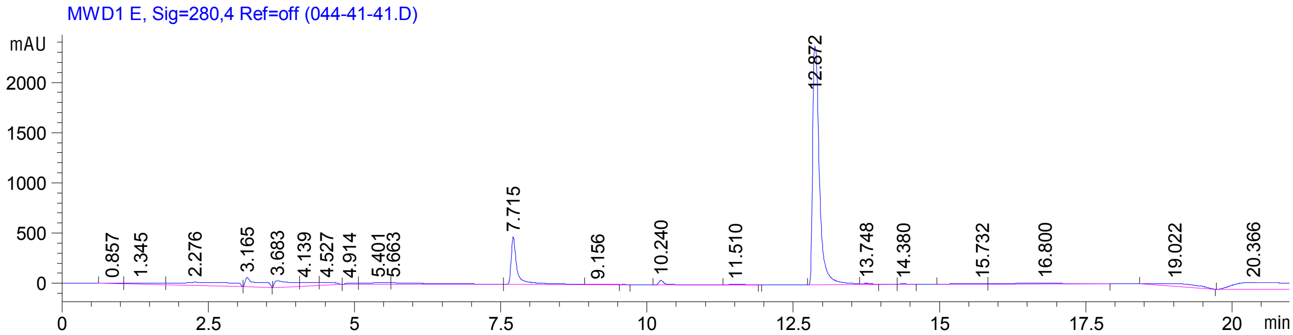

Figure S54 – Biocatalysis: HPLC chromatogram of filtered supernatant for *E. coli* MC4100 pSTB7 cultures following incubation in the presence of 0.05 mg/mL of **P1-*mod*-OctA** for 24 h, followed by incubation with reaction buffer for another 24 h.

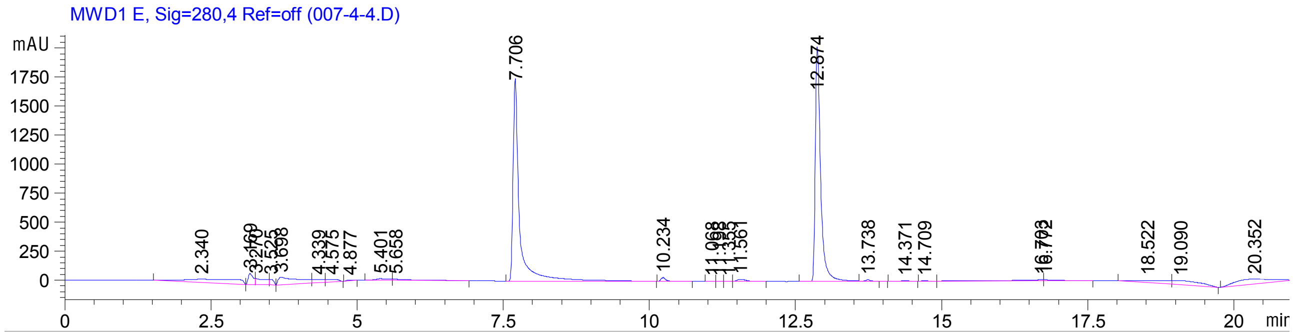

Figure S55 – Biocatalysis: HPLC chromatogram of filtered supernatant for *E. coli* MC4100 pSTB7 cultures following incubation in the presence of 0.05 mg/mL of **P1-*mod*-2NphA** for 24 h, followed by incubation with reaction buffer for another 24 h.

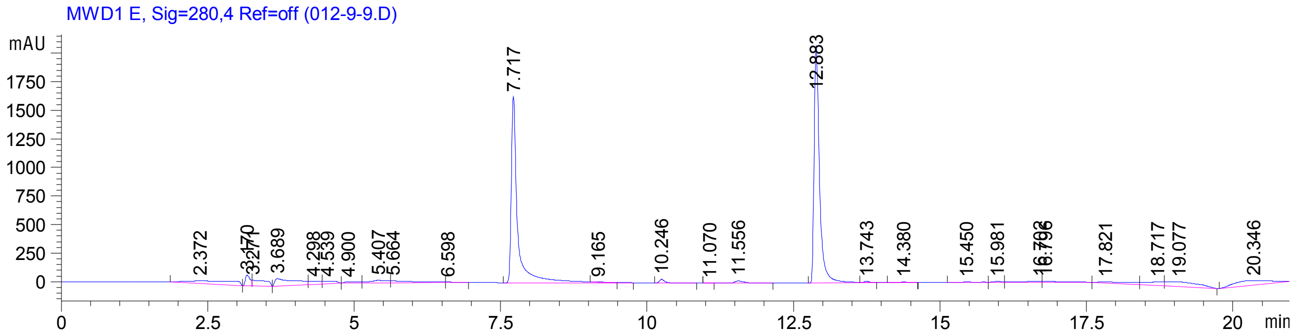

Figure S56 – Biocatalysis: HPLC chromatogram of filtered supernatant for *E. coli* MC4100 pSTB7 cultures following incubation in the presence of 0.05 mg/mL of **P1-*mod*-9AntA** for 24 h, followed by incubation with reaction buffer for another 24 h.

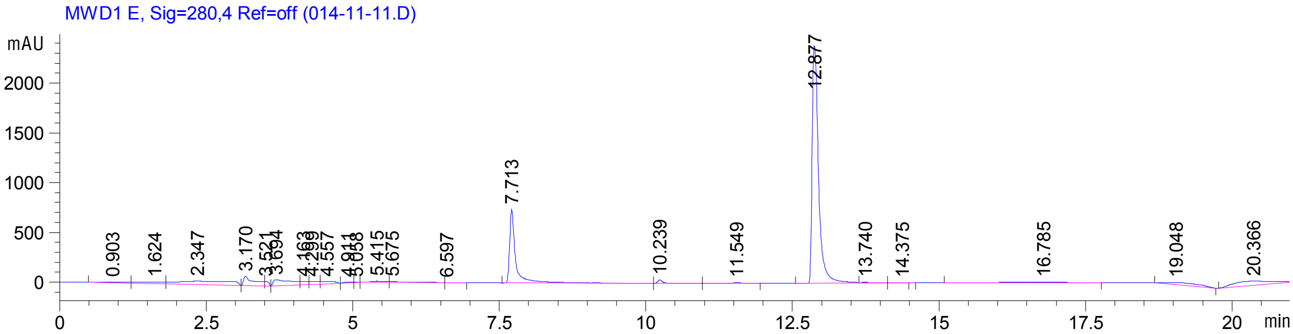

Figure S57 – Biocatalysis: HPLC chromatogram of filtered supernatant for *E. coli* MC4100 pSTB7 cultures following incubation in the presence of 0.05 mg/mL of **P1-*mod*-1PyrA** for 24 h, followed by incubation with reaction buffer for another 24 h.

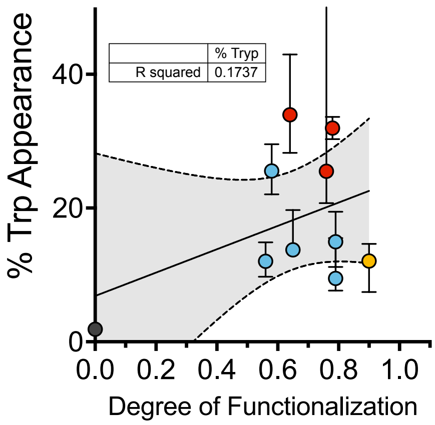

Figure S58 – Correlation of degree of functionalization with 5-fluorotryptophan production: Percentage of 5-fluorotryptophan appearance as a function of degree of functionalization, for *E. coli* MC4100 cultures following 48 h of incubation in 100 mM phosphate buffer at pH 7 in the absence (lower white dot) and presence of 0.05 mg/mL of **P1** (black solid dot) and functional polymers **P1-*mod*-aldehyde** (coloured dots), followed by incubation with reaction buffer for another 24 h. Median and 25th to 75th percentiles shown for at least 4 replicates.

9. Metabolic Activity: Reduction of resazurin to resofurin

Polymer-cell suspensions (MC4100) were prepared as detailed in section 3 above. Briefly, the pellet was resuspended in 0.1 M KH_2PO_4/K_2HPO_4 so that an OD_{600} of 0.2 was reached. Polymers were then added to the culture at 0.1 mg/mL, the culture supplemented with an equal volume of standard M63 minimal media. For this assay, cells were resuspended to a final volume of 1 mL in eppendorf tubes and incubated at 30 °C, 150 rpm for 24 and 48 h respectively. Aldehyde-cell suspensions were also prepared. After incubation, the samples were centrifuged (12000 g, 5 min) and the supernatant media discarded. The samples were then resuspended in 1 mL PBS, vortexed for 20 s and sonicated (30 s x 2). 190 µL of each sample was transferred into a well of a 96-well plate and incubated for 30 min at 37 °C. 10 µL of resazurin was then added to give a final concentration of 0.05 mM resazurin. After 30 min incubation at 37 °C in the dark, resorufin fluorescence (590 nm) was measured.
